# Supplementary material for: Isotopic evidence for initial coastal colonization and subsequent diversification in the human occupation of Wallacea
Source: Nat Commun. 2020 Apr 29;11:2068. doi: 10.1038/s41467-020-15969-4 (PMC7190613; doi:10.1038/s41467-020-15969-4)
Supplement: Supplementary file 1 — Supplementary Information [file 41467_2020_15969_MOESM1_ESM.pdf]

Supplementary Information for

Isotopic evidence for initial coastal colonization and subsequent diversification in the human occupation of Wallacea

Roberts et al.

This PDF file includes:
Supplementary Note 1: Site background and chronostratigraphic information
Supplementary Figures 1-16
Supplementary Tables 1-12
Supplementary References

Supplementary Notes

Supplementary Note 1: Site background and chronostratigraphic information

For all of the sites, absolute age determinations were obtained through radiocarbon dates and, in the case of Asitau Kuru, additional Optically Stimulated Luminescence (OSL) dates. All radiocarbon dates were calibrated using the IntCal13 calibration curve for charcoal, and the Marine13 calibration curve for shell [1]. No delta R value was used to adjust the marine shell dates because a local value has not been established for this region and its affect is on sub-millennial timescales so it would be unlikely to alter any of the broader phasing used here (Rachel Wood pers. comm.). The majority of this chronometric and stratigraphic information has already been published as noted below.

Timor

Asitau Kuru (formerly Jerimalai)

The site of Asitau Kuru (formerly named Jerimalai) has been subjected to repeated multidisciplinary investigation since 2005. The limestone shelter is located 75 m asl southeast of Tutuala on the eastern coast of East Timor (GPS: 8'24.84' S, 127'17.50' E) [2-5]. The local vegetation consists of mesophyll vine and evergreen forests. Excavations of three 1 x 1 metre trenches has uncovered approximately 1.8 metres of stratigraphic sequence of human occupation dating back to ~46,000 years ago (Supplementary Figure 1; Supplementary Table 1). As summarized by Shipton *et al.* [3] there are now a total of 74 absolute age determinations from Asitau Kuru including eight Optically Stimulated Luminescence (OSL) dates and a combined total of 66 radiocarbon dates on charcoal and marine shell (Supplementary Table 1). The samples from this study come from adjacent Squares B and C that we divide into chronological Phases based on stratigraphy and age estimates (as per [3]).

The sequence is divided into three broad chronological Phases: a Neolithic occupation in the last 4000 years, spanning layers 1-2, (Square B spits 1-14, Square C spits 1-16); an early to middle Holocene occupation (10,000-4000 years ago) spanning layers 3-5 (Square B spits 15-42, Square C spits 17-41); and a Pleistocene occupation going back to around 45,000 years ago, spanning layers 6-8 (Square B spits 43-67, Square C spits 42-60), which may be further subdivided into the Terminal Pleistocene layer 6 (Square B spits 43-52, Square C spits 42-50, ~20,000-12,000 years ago), and the pre-LGM layers 7-8 (Square B spits 53-67, Square C spits 51-60, ~46,000-29,000 years ago). As noted by Shipton *et al.* [3], the integrity of these chronological Phases is demonstrated by the absence of pottery below Layer 2, the absence of exotic lithics and *Nassarius* beads below Layer 5, and distinct patterns between the phases in terms of proportions of natural versus knapped stone clasts and bone versus shell zooarchaeological material [3].

The distribution of the dates, as well as lithic analysis, has been used to argue that occupation of the site begins with low intensity Pleistocene occupation prior to a possible hiatus just before the Last Glacial Maximum and a resumption of low intensity, sporadic occupation in the Terminal Pleistocene in Timor-Leste [3, 6]. More than 80% of the Asitau Kuru zooarchaeological assemblage is composed of marine

animals, with a shift from dominance of Scombridae and Carangidae in the Late Pleistocene and Early Holocene to increasing numbers of Scaridae, Ballistade, Serranidae, and Acanthuridae in the Middle and Late Holocene, alongside increased fish relative to shellfish consumption in general [2-3]. Bones of marine turtles are also present.

In terms of terrestrial fauna, two genera of giant rats (*Coryphomys* and Glover's [7] 'Genus A') have been thus far recognized from this site, as well as smaller rodents such as *Melomys* spp., *Komodomys* spp., and *Rattus exulans.* These taxa are represented by burnt and unburnt remains, suggesting some level of human consumption, although incidental burning not associated with consumption cannot be ruled out. Other than rodents, the site has preserved small numbers of birds, including an extinct crane, extant pigeons, pheasants, quails and songbirds [8]. Megabat remains were also recovered (*Pteropus* and *Dobsonia*), and reptiles such as geckos, skinks, and snakes are present in most spits.

There has been debate as to whether the earliest inhabitants of Asitau Kuru were undertaking pelagic, offshore fishing [9]. The procurement of tuna, as well as the presence of one of the world's oldest fishing hooks crafted from shell seems indicative [2], though definitions of pelagic species and environments are disputed [10]. The presence of marine shell beads throughout the sequence, some of which are covered in ochre, has also been used as evidence for early jewelry and socially oriented personal display in Wallacea [4]. Nevertheless, while a social and economic focus on marine environments, and perhaps complex maritime technologies, has been assumed, it has remained impossible to directly test overall human reliance on marine versus terrestrial resources, with the possibility that missing archaeobotanical data (perhaps due to taphonomic bias against starchy plants), and seasonal use of the site is causing an overestimation of human reliance on marine resources.

Lene Hara

Lene Hara (GPS: 8' 24.35' S, 127'17.58' E) is a large cave located less than a kilometre away from Asitau Kuru and within a kilometre of the current coastline [4]. Today the cave is surrounded by secondary regrowth forest and remnants of old garden walling can be seen along the forest tracks leading to the cave. Lene Hara has been systematically investigated since 2000 [11-14], and has produced a stratigraphic sequence dated back to *c.* 39,000 cal BP in Square A in the southern chamber [13]. The chrono-stratigraphic sequences of the different excavation areas in the cave are published in detail in O'Connor *et al.* [13] and show that there has been significant variation in net sediment accumulation across the cave floor. In this study, we analyzed one human third molar from Square F in the northern chamber of the cave, excavated in 2002.

Square F produced a stratigraphic sequence down to *c.* 2 metres without reaching bedrock, with an exclusively Holocene sequence. The basal date from Square F in Spit 43 has an age estimate of *c.* 11,000 cal. BP [13] (Supplementary Figures 2 and 3; Supplementary Table 2). From the Square F excavation, a number of marine shell beads were recovered, as well as a fish hook dated to the early Holocene (*c.* 10,500

cal. years BP (Supplementary Table 2) [4, 14]. The tooth from Lene Hara F comes from Spit 37/38 and is dated to approximately 7,000 cal. years BP [13].

Currently, no detailed zooarchaeology has been undertaken at the site, making isotopic analysis of relative reliance on terrestrial versus marine resources essential. Nevertheless, a preliminary species list has been prepared for this excavation. Introduced species to Timor, such as cuscus, pig, and dog, are restricted to the Neolithic layers. Rodents identified from this square include Glover's giant rat 'Genus A', *Komodomys* spp., *Melomys* spp., and *Rattus* spp. Glover's [7] giant rat 'Genus B' has also been recovered from the nearby B square.

Matja Kuru 1 and 2

The cave sites of Matja Kuru 2 (GPS: 8'24.88' S, 127'07.42' E) and Matja Kuru 1 (GPS: 8'24.87' S, 127'07.36' E) neighbor each other within a limestone ridge northeast of Poros village, and just north of Lake Ira Lalaro on the island of Timor-Leste. Local vegetation is dominated by mesophyll vine and evergreen forests at the ridgeline, with an ecotone transition to open savannahs and grasslands towards the lake and village. Both sites sit *c.* 370 m a.s.l. and *c.* 8 km away from the northern coast of the island. These sites were excavated in 2001 and 2014. In 2001 a 1 x 1 metre trench (Square D) was excavated at Matja Kuru 2 and a 1 x 2 metre trench (Squares A and AA) at Matja Kuru 1 73 (Supplementary Figures 4-6). In 2014, Matja Kuru 2 was re-opened in order to extend the depth of the original excavation to bedrock, and enlarge the excavation area. A 3 x 2 metre area was excavated, taking in the original Square D, and including five new squares, DD, C, B, BB and AA, of which Squares C and B have been sampled here (Supplementary Figures 5-6). The trenches of the 2001 and 2014 excavations at Matja Kuru 2 and Matja Kuru 1 have now been intensively dated using radiocarbon dating of charcoal and marine shell, returning ages that, together, span *c.* 40,000 years ago to *c.* 3,000 years ago [4] and unpublished data. Overall the sequences are believed to have good stratigraphic integrity, and with the exception of a few sherds, pottery is restricted to the late Holocene [15].

Phasing produced for Matja Kuru 2 based on archaeological and chronometric information (Supplementary Table 3) is as follows: Spits 1-13 in both squares B and D date to the late Holocene (3,500-2000 years ago); Spits 16-46 of Square B and spits 15-35 of Square D represent an early Holocene (11,000-9,500 years ago); and Spits 47-78 of Square B and Spits 36-42 of Square D cover a Late Pleistocene period (c. 40,000-31,000 years ago). For Matja Kuru 1 Square AA there are two principal phases: a middle Holocene phase from spits 7-20 (~4,500-6,000 years ago) and a Terminal Pleistocene phase from spits 21-25 (~11,000-16,000 years ago). Individual human teeth were recovered from Matja Kuru 1 Square AA, spits 8, 11, 16, and 17, which date to the middle Holocene, while two teeth were recovered from the Terminal Pleistocene initial occupation. From Matja Kuru 2 two human teeth derive from D31 and D33, associated with early Holocene dates.

The material from the 2014 excavation at Matja Kuru 2 is still under analysis, however, excavation units (spits) can be broadly correlated with the units from D at the same approximate depth, and the dating that has been carried out supports the view that the chronology and occupation sequences of the adjoining squares are

broadly comparable. Marine shell in the lowest datable spit of the 2014 extension of Square D, Spit 52, returned an age of 34,650 ± 418 BP (Wk-41364) or *c.* 39,500-37,500 cal. BP. Our sample includes two human teeth from the 2014 excavation from Squares C (C16) and B (B40). The human tooth from B40 is bracketed by marine shell dates from B16 of 9414 ± 23 BP (Wk-41353) and B42 9391 ± 20 BP (Wk-41122). Based on comparison with the adjoining B square stratigraphy and depth below surface, the human tooth from C16 should be of the same age as that from B16, falling into the Terminal Pleistocene to mid Holocene Phase (Supplementary Table 3).

Matja Kuru 1 and 2 contain stone artefacts, shell beads, ochre and the bones of giant rats, reptiles, marine shellfish and other marine fauna throughout their sequences. Occupation of the sites has been interpreted as follows. It is thought that Matja Kuru 2 was a campsite used by mobile foragers approximately 40,000-30,000 years ago. During this time, resources from the freshwater lake, Lake Ira Lalaro, were exploited, as well as marine resources, brought back to the cave from the coast. A remarkable bone artifact with adhering mastic and deep notches, interpreted as the haft end of a projectile point, has been recovered from Spit 43 and is dated to *c.* 36,000-34,000 cal. years BP [16]. Matja Kuru 2 was then seemingly little used, or abandoned, during the Last Glacial Maximum, perhaps due to low water levels in Lake Ira Lalaro.

In the Terminal Pleistocene and Holocene, initial occupation is identified at Matja Kuru 1 (from *c.* 16,000 years ago) and Matja Kuru 2 is reoccupied (from *c.* 13,000 year ago). Based on subsistence remains and stone artefacts deposited, habitation at Matja Kuru 2 appears less intense during this time, and there is no evidence for use of the cave during the Middle Holocene [17]. Occupation appears to have switched to Matja Kuru 1 at this time (*c.* 6,500-4,500 years ago), with a peak in the deposition of bone and shellfish. Matja Kuru 2 is used episodically in the late Holocene from *c.* 4000 years ago until Historic times, including a dog burial dated to 2,921-3,075 cal. years BP [18].

Terrestrial fauna recovered from Matja Kuru 1 and 2 are still being analysed, however, it includes introduced species (*Phalanger, Macaca, Sus, Cervus, Canis*) and the potentially expatriate shrew *Crocidura,* all restricted to Holocene layers. Giant rats include all four giant rat genera (*Coryphomys,* Glover's Genera 'A', 'B', and 'C'), while smaller rats include large and small forms of *Komodomys, Melomys,* and *Rattus*. These are found throughout the sequences with the exception of *Rattus exulans,* which is restricted to the top 11 spits in Matja Kuru 1. Birds recovered from Matja Kuru 1 include ducks, pheasants, pigeons, rails, quails, and small songbirds [74].

Alor

Makpan

Makpan is a large lava tube cave (GPS: 08°25.931'S, 124°21.230'E) with a southward facing entrance that sits *c*. 37.5 metres a.s.l and *c*. 386 metres inland from the shore of Alor's south coast. It is formed within the upper Tertiary volcanic Alor Formation [19]. The local environment is largely dry, open grassland. The entrance to Makpan is over 8 metres high with the ceiling height steadily declining inside the caves' *c*. 94 metre extent, due to a gently upward sloping floor.

The cave was excavated in 2016. A main 2 x 2 metre area, excavated as four contiguous squares A, B, C, & D, was opened inside the mouth of the cave, within the protection of the dripline (Supplementary Figures 7-8). This excavation extended to a depth of 1.5 m before instability in the deposit necessitated shoring. From this point only a single 1 x 1 metre area, Square B, was continued (Kealy et al., forthcoming). Square B reached an additional *c.* 2 metres, making a maximum total excavation depth of 3.5 metres for the Makpan main trench. Sterile beach sand was encountered in parts of Square B in the bottom *c.* 20 cm of the trench with excavation discontinued once all cultural deposits had been removed and beach sand exposed across the entire square. A second smaller excavation area was positioned deeper inside the cave beneath a modern-day owl roost. This 1 x 1 metre trench was excavated to a maximum depth of 1 m with the lowest layer dated to *c*. 7.9 – 7.6 ka [20]. The owl roost deposit was excavated to provide a sample of non-human derived fauna for comparison with that recovered from the main trench (Supplementary Figure 9).

Extensive radiocarbon dating has demonstrated Makpan to be the oldest known human occupation site on the island of Alor with a date of *c.* 40,000 cal BP from the basal layer of the cultural deposit (Supplementary Table 4). The Makpan sequence can be divided into four chronological phases. A Neolithic occupation phase begins *c.* 3.4 ka and is represented by Layers 1 to 4 (Spits 1-8 for all Squares). Below this is an Early - Middle Holocene phase (10 – 7.5 ka) that spans Layers 5 to 9 (Spits 9-19 for all Squares). This is underlain by a Terminal Pleistocene to initial Holocene phase which includes an extremely dense shell midden dated to between 15 ka and 11 ka (Layers 10 – 17) (Squares A, B, C, & D, spits 20-23, and Square B spits 24-57). The lowermost Late Pleistocene phase begins at *c.* 40 ka and continues with episodic occupation through until *c*. 21 ka, spanning spits 58–68 of Square B.

The main excavation at Makpan recovered an assemblage of stone, shell, and coral artefacts, including a variety of shell bead and fishhook types. Pottery was found in the Neolithic occupation phase. Subsistence remains included a diversity of marine invertebrates, crustacea, fish, reptiles, and small and large rodents. Terrestrial fauna includes the giant rat *Alormys aplini*, smaller rats *Rattus* spp., and the lizard *Varanus* sp. It is in the more recent layers of the Middle Holocene phase at Makpan where the remains of a juvenile human burial (stratigraphic sample number: HT-D10) were recovered between spits 12 – 10 of square D (Layer 5). Direct radiocarbon dating of the tooth enamel obtained an age of 7.95 – 7.8 ka cal BP (7041 ± 36 ANU 56516), contemporaneous with charcoal and shell dates from Layer 5 (Supplementary Table 4).

After a *c.* 5,000-year hiatus in the record Makpan sees increased sedimentation and occupation during the Terminal Pleistocene-Early Holocene phase, culminating in the *c.* 12 – 11 ka shell midden deposit. It is from this deposit that our second human isotope sample (stratigraphic sample number: HT-B57) was obtained from an isolated tooth. The exceptionally dense shell midden, which is also rich in shell artefacts, the remains of other marine invertebrates, fish and stone tools, indicates that not only was this the period of densest human occupation at Makpan, but that this community intensively exploited marine resources for subsistence. As a result of slower sedimentation rates and compaction from the dense shell midden deposit above, the lowest *c.* 0.5 m of the Makpan deposit in Square B has become a single time averaged layer for the 20,000-year period between 40 – 21 ka.

Tron Bon Lei

Tron Bon Lei encompasses two adjacent rockshelters (08°26.077'S, 124°33.826'E) located on the southwest coast of the island of Alor near Lerabain village. They are formed in a volcanic ridge located *c.* 33 metres a.s.l. and 160 metres inland from the shore. Today, the local environment is comprised of open schlerophyll woodland and grasslands [21]. The Tron Bon Lei shelters, as well as the other shelters and caves in this part of Alor, are formed from basaltic to andesitic volcanic deposits and their floors consist of unconsolidated sediment and volcanic boulders of the same composition as the walls [22]. Investigation of Tron Bon Lei rockshelter in 2015 involved the excavation of three 1 x 1 test pits in the two adjacent shelters with the same name [22]. Two trenches (Squares A and C) were located in the west-facing shelter while a third (Square B) was located in a southerly facing shelter *c.*15 metres to the east [21, 23] (Supplementary Figures 10-12). In 2018, two new excavation Squares, D and E, were opened adjacent to Square B.

Square C (Supplementary Figure 11) reached bedrock at a depth of 0.7m, but the remains of two individuals (burial C1 and burial C2) were recovered from near the base (Layer 4) [24]. Direct U-Th and associated C-14 dating suggests an age of around 7.5 ka for these burials. An older age of *c.* 17 ka is possible based on an OSL date and is consistent with a late uptake of uranium by the bone, however, the OSL provides an age of sediment inside the skull which may represent the sediment the burial was interred into. Square B was excavated down to bedrock at a depth of 3.2 metres [24]. Thirteen stratigraphic layers, which included hearths, shell midden layers, white ash lenses, and a secondary burial, were identified for Test Pit B, and were dated using 24 radiocarbon dates from charcoal and marine shell [21-23, 25].

The available radiocarbon dates from Square B have enabled its division into three major occupation Phases [21-22] (Supplementary Table 5). The youngest phase incorporates Layers 1 to 5 in a Late Holocene period (4,090-3,010 cal. years BP). A Terminal Pleistocene to Middle Holocene Phase includes Layers 6-11 (12,545-7,440 cal. years BP) and an older Late Pleistocene Phase of occupation in Layers 12 and 13 is dated to 21,000-18,890 cal. years BP [21-23]. The adjacent Squares D and E produced two burial features (overlapping both squares), cut from the stratigraphic levels of Square B Layers 6 (burial D1) and 10 (burial D2). Multiple sets of human remains were encountered in Square B, with an articulated skeleton associated with marine shell fishhooks in Layer 11 [26]. Radiocarbon dating of one of the fishhooks, as well as

charcoal associated with the burial, indicate a burial age between 12,000 and 11,000 years ago. The six fishhooks found with this burial (an additional one was recovered when the remainder of the skeleton was excavated from Square D) are the only ones that have been found at Tron Bon Lei and suggest the cultural importance of marine resources to individuals living at the site at this time [22-23, 25]. We sampled this individual as well as the two overlying burials from Square D, and burials 1 and 2 from Square C.

A zooarchaeological analysis of Square B indicated the presence of predominantly marine fauna with fishing for carnivorous and herbivorous reef fish fluctuating in frequency through the period of occupation, apparently in association with changes to marine ecologies and cultural practices, particularly between the Terminal Pleistocene and Middle Holocene [22]. Marine turtle and terrestrial vertebrates (including small rodents, lizards, snakes, frogs/toads, fruit bats, and microbats) were also identified [22]. Overall, the terrestrial faunal assemblage has been interpreted as largely the result of barn owl predation of microfauna. Human predation of fish and other marine fauna constitute the bulk of the zooarchaeological assemblage, with larger terrestrial fauna such as pythons and fruit bats also potentially contributing in a small way to subsistence [21].

Regional Phasing used in this study

As noted in the main text, to display and compare our results most effectively we have sought to divide the faunal and human data into a series of Phases of occupation on Timor and Alor, respectively, based on the stratigraphic and chronometric information for each site presented above (Supplementary Tables 6 and 7). For Timor, and the sites of Asitau Kuru, Lene Hara, Matja Kuru 1, and Matja Kuru 2 (Timor-Leste) this system includes four broad phases: a Late Pleistocene pre-LGM Phase (46,000-29,000 years ago), a Terminal Pleistocene Phase (20,000-11,001 years ago); an Early and Middle Holocene Phase (11,000-4,001 years ago; and a Late Holocene Neolithic Phase (4000-0 years ago). For Alor, and the sites of Makpan and Tron Bon Lei, the phasing system includes three broad Phases: a Late Pleistocene pre-LGM Phase (40,000-21,000 years ago), a Terminal Pleistocene to Middle Holocene Phase (15,000-7,400 years ago); and a Late Holocene Neolithic Phase (4000-0 years ago). The specific dates for the human samples within these broader phases are discussed in the main text where appropriate.

Our phasing for the two islands enables us to compare the subsistence of the earliest human occupants of Wallacea so far discovered, humans responding to sea-level and environmental changes at the Terminal Pleistocene-Early Holocene transition [5], and postulated cultural, population, and subsistence shifts with the arrival of the Neolithic. Following apparently relatively sporadic Late Pleistocene human occupation prior to the Last Glacial Maximum, the very end of the Pleistocene of Wallacea has been argued to witness greater social connectivity and demographic expansion, as evidenced by inter-island obsidian transfers and the occupation of very small islands [27-28]. This is followed by ground shell axe technology and the formation of large middens in the early and middle Holocene. These changes have been linked to increasingly intensive regional occupation during a period of rising and then stabilizing sea level [6, 29].

The most comprehensive study on subsistence strategies of humans on Timor suggested that they were opportunistic and highly adaptable, switching their protein resources depending on local availability and environment [30]. However, limited palaeoenvironmental information exists for this region during the period of interest. Today, less than 15% of the primary forest cover on Timor remains, however, original cover was likely significantly more extensive, consisting of evergreen and semi-evergreen forests, deciduous forests, and sclerophyll vegetation [31]. Pleistocene Alor is perhaps even more poorly known, however, it probably resembled Timor's lower topographic areas given the geographical proximity of the two islands. Evidence for C_3 vegetation from at least the beginning of the Holocene is provided by isotopic analyses of the giant rat from Alor [20]. Increased aridity and reduced rainfall from about 2,500 years ago [32] as well as anthropogenic deforestation since the late Holocene probably significantly reduced tree cover on both islands [20].

Supplementary Figure 1. Stratigraphic sequence of Asitau Kuru (Square B) (Timor-Leste) and location of human samples (HT=Human Tooth).

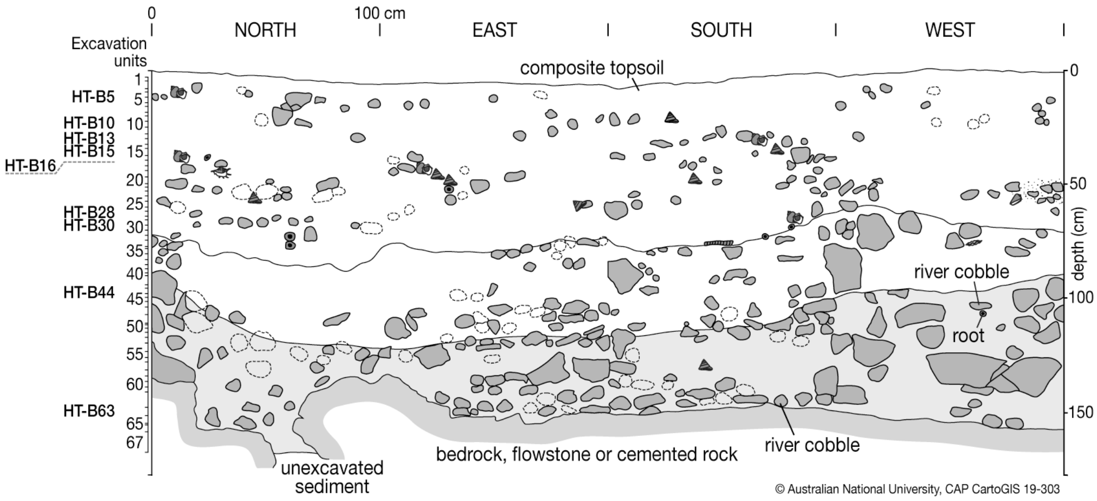

Supplementary Figure 2. Location of Lene Hara Square F (Timor-Leste) within the site.

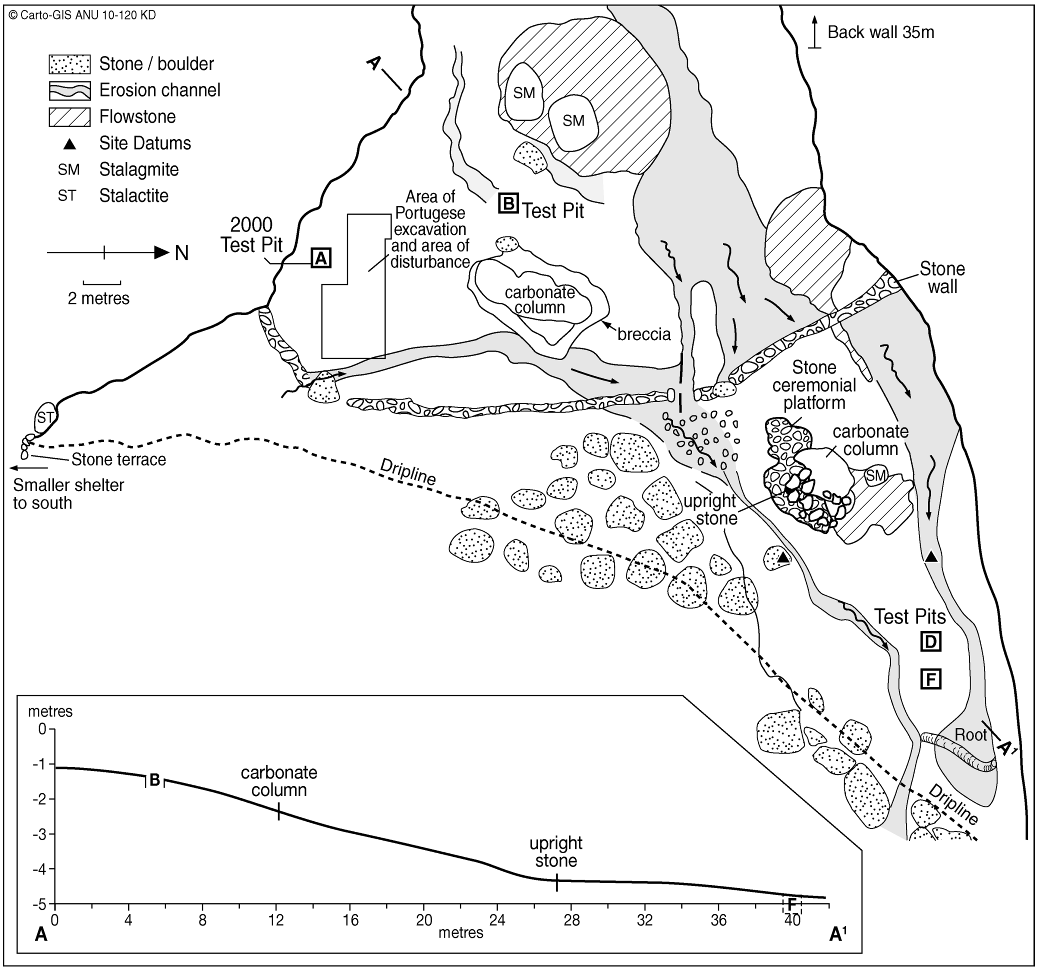

Supplementary Figure 3. Stratigraphic sequence of Lene Hara (Square F) (Timor-Leste) and location of human sample (HT=Human Tooth).

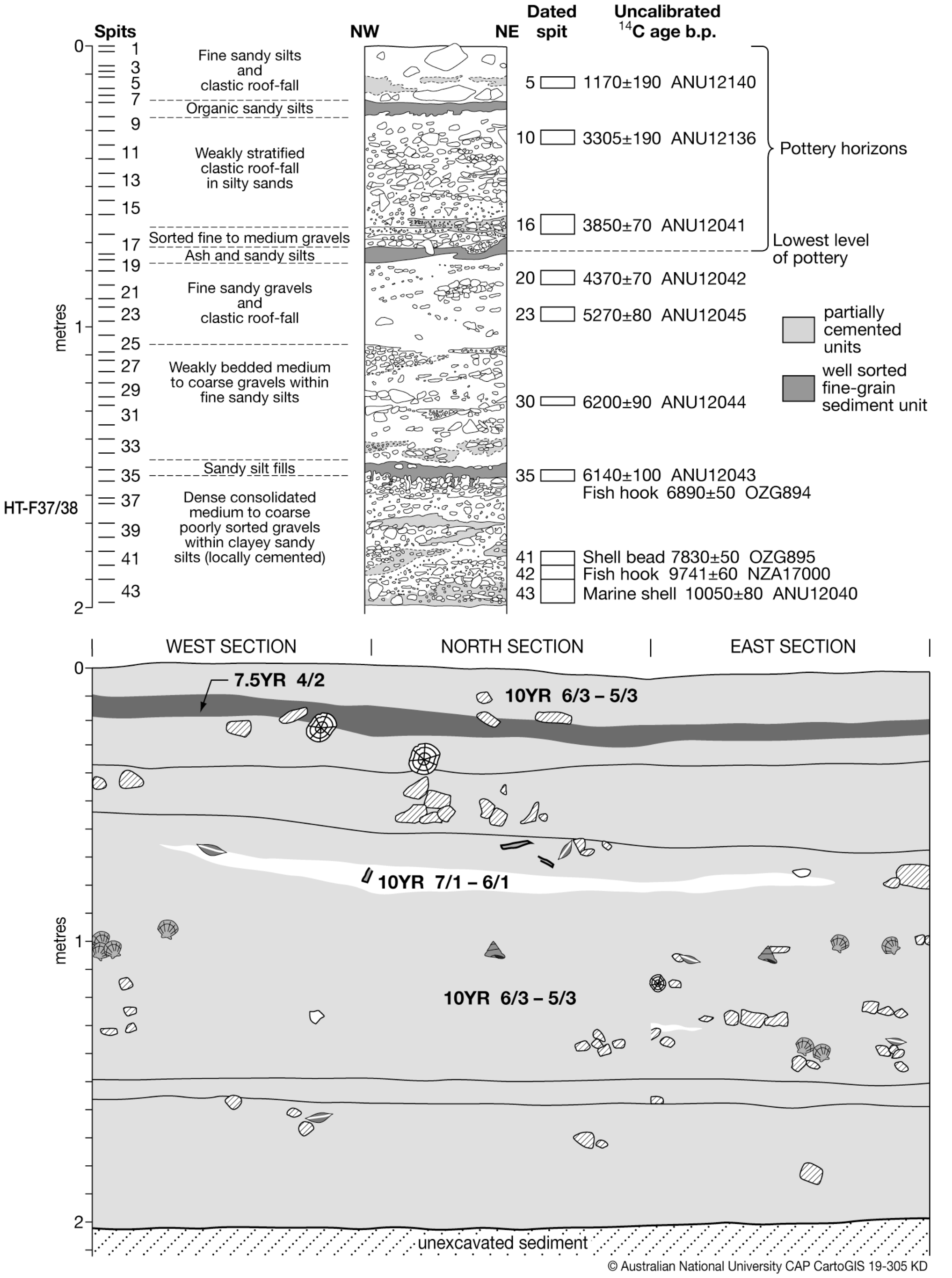

Supplementary Figure 4. Stratigraphic sequence of Matja Kuru 1 (Square AA) (Timor-Leste) and location of human samples (HT=Human Tooth).

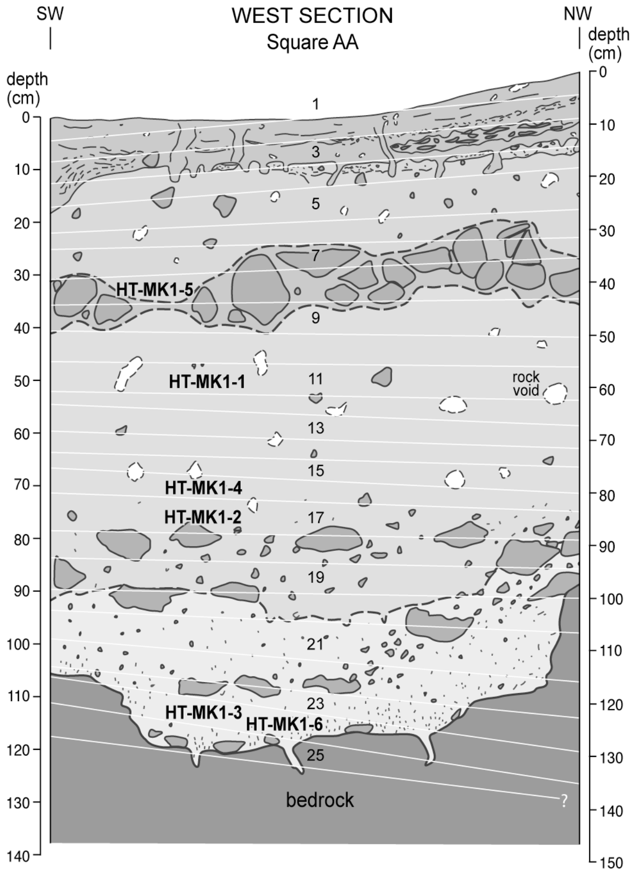

Supplementary Figure 5. Stratigraphic sequence of Matja Kuru 2 West section (Squares B and C) (Timor-Leste) and location of human samples (HT=Human Tooth).

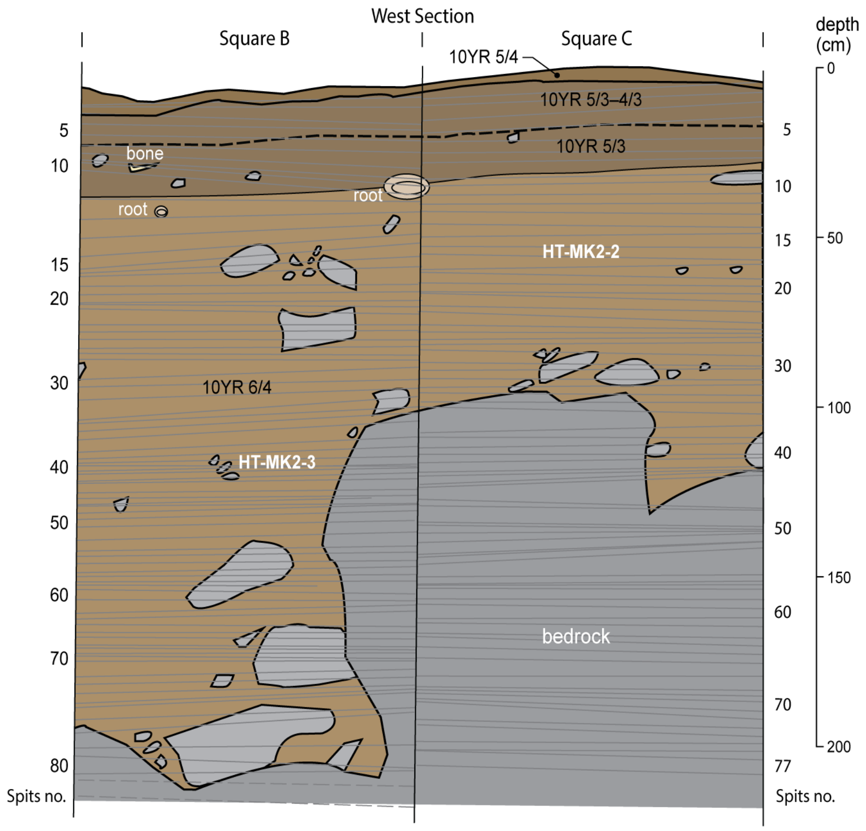

Supplementary Figure 6. Stratigraphic sequence of Matja Kuru 2 East section (Square D) (Timor-Leste) and location of human samples (HT=Human Tooth).

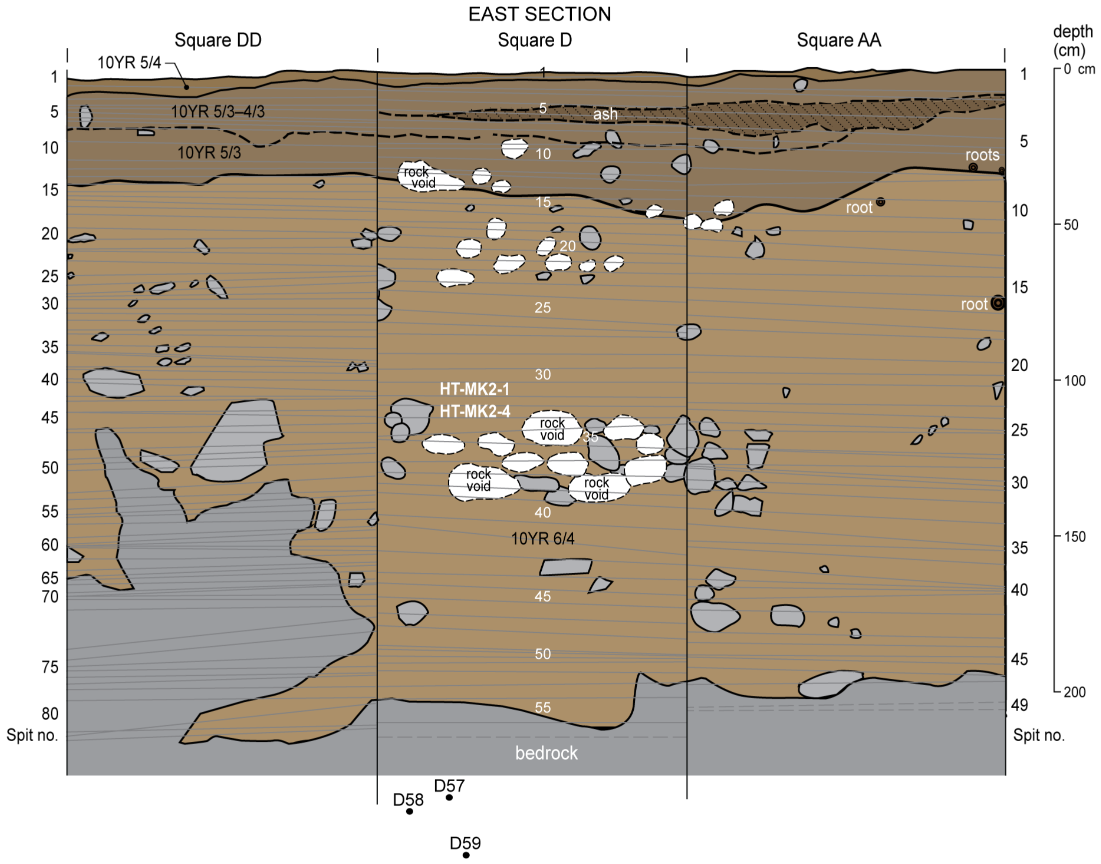

Supplementary Figure 7. Stratigraphic sequence of Makpan (Square B) (upper) (Alor) and location of human samples (HT=Human Tooth).

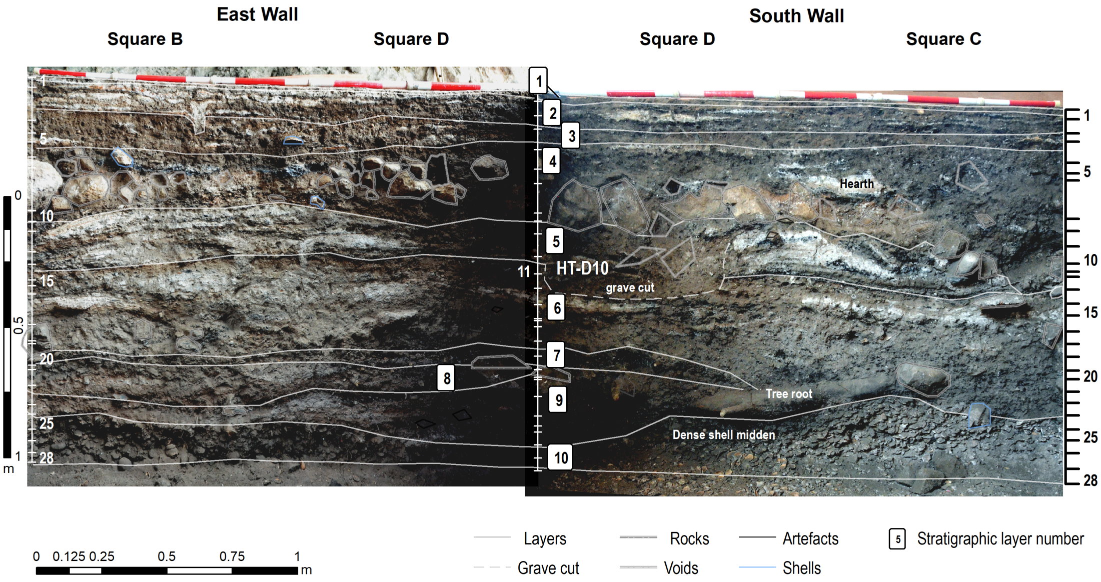

Supplementary Figure 8. Stratigraphic sequence of Makpan (Square B) (lower) (Alor) and location of human samples (HT=Human Tooth).

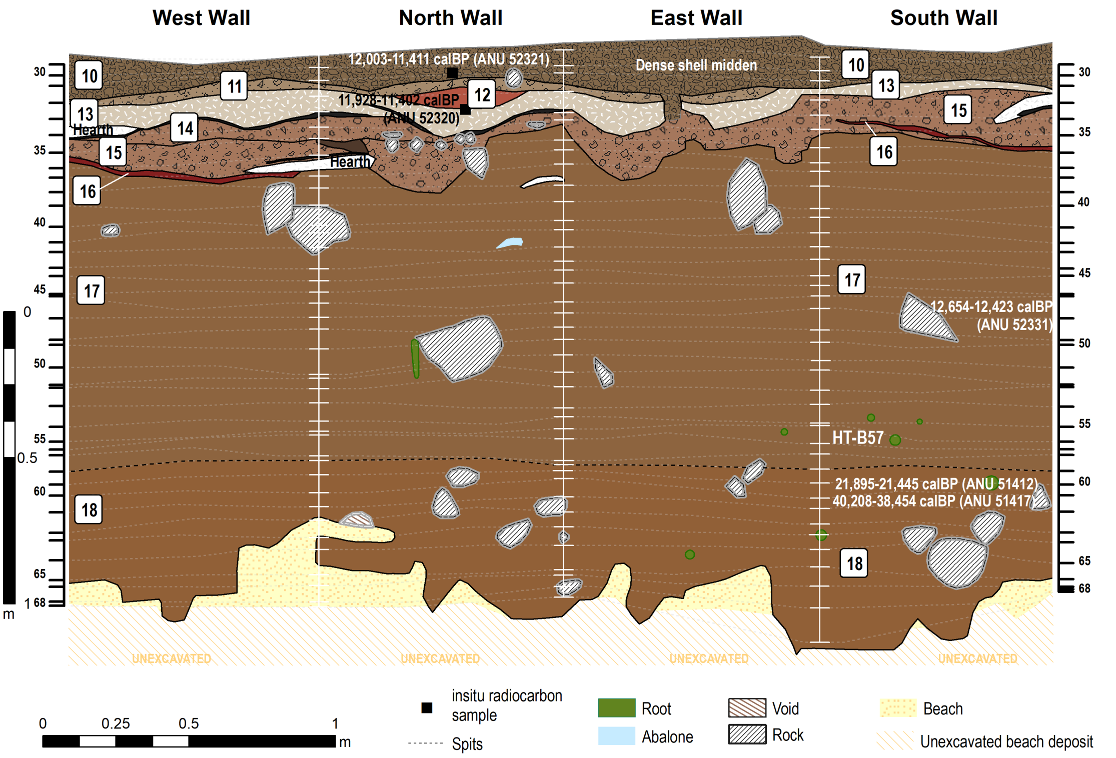

Supplementary Figure 9. Stratigraphic sequence of Makpan owl roost (Alor).

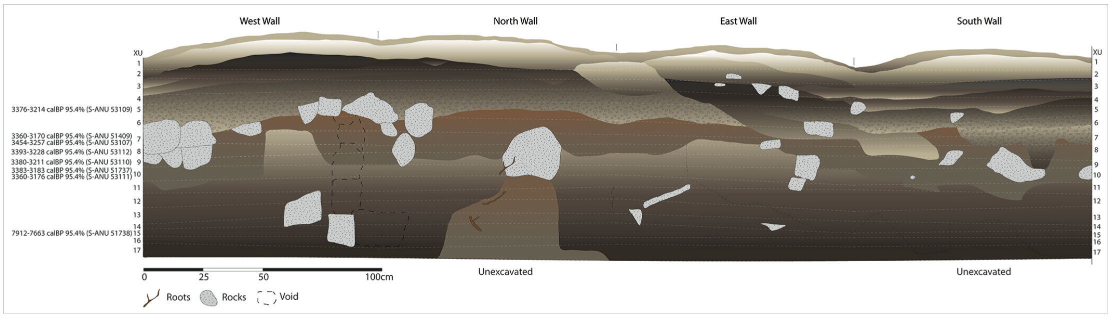

Supplementary Figure 10. Stratigraphic sequence of Tron Bon Lei (Square B) (Alor) and location of human sample (HT=Human Tooth).

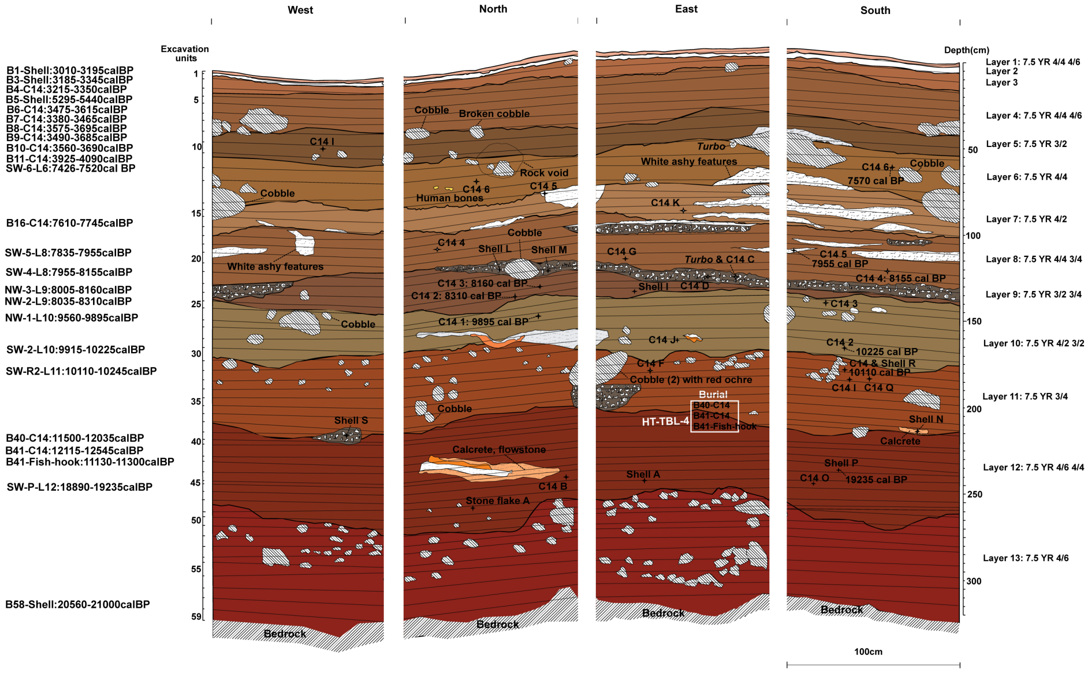

Supplementary Figure 11. Stratigraphic sequence of Tron Bon Lei (Square C) (Alor) and location of human sample (HT=Human Tooth).

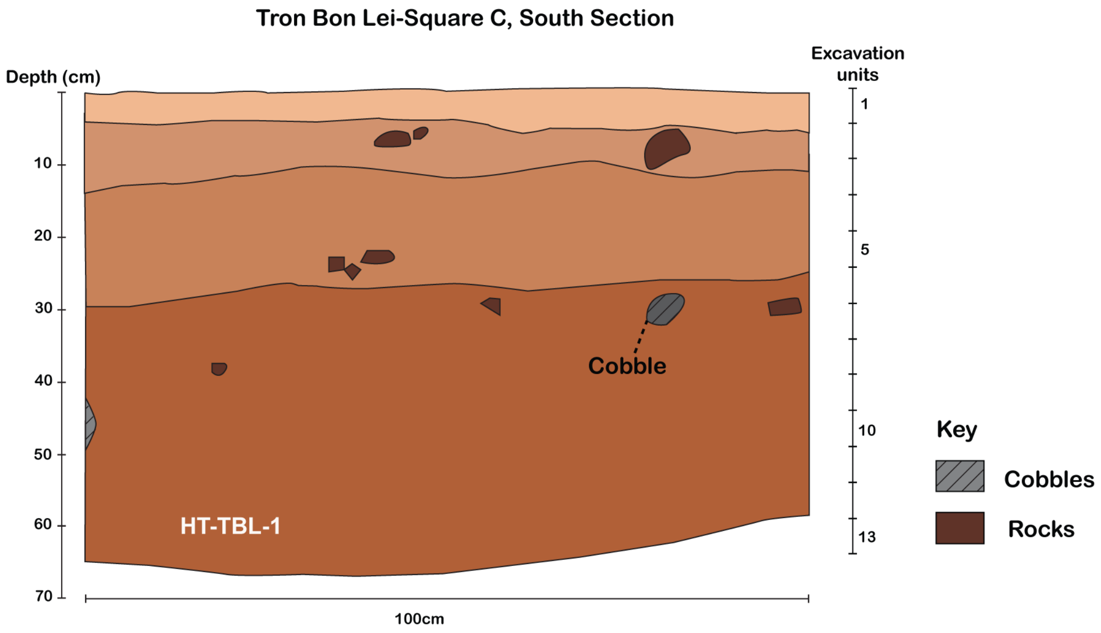

Supplementary Figure 12. Stratigraphic sequence of Tron Bon Lei (Squares D and E) (Alor) and location of human samples (HT= Human Tooth).

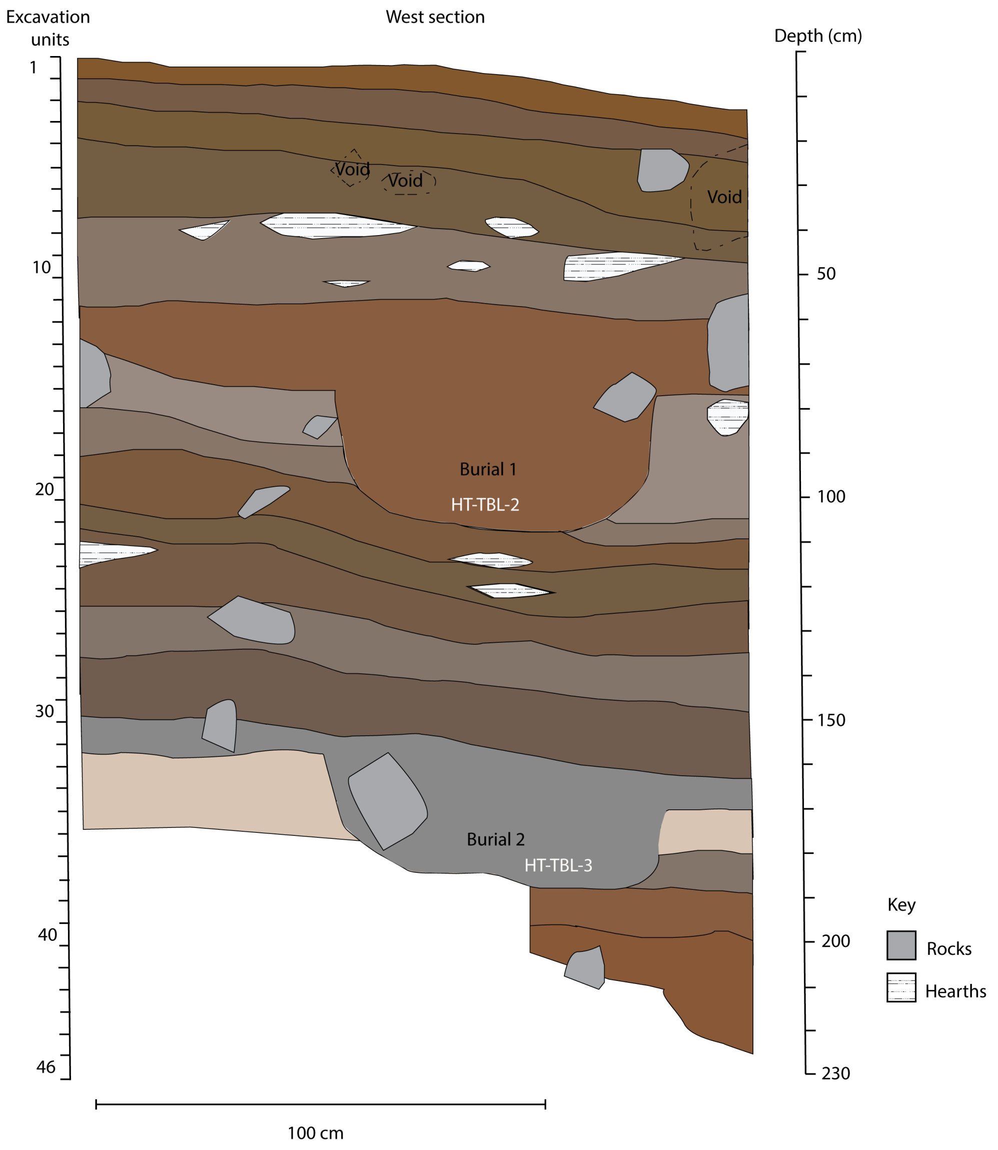

Supplementary Figure 13. Combined stable carbon ($\delta^{13}C$) and oxygen ($\delta^{18}O$) isotope data from terrestrial and marine faunal tooth enamel samples analyzed in this study from the island of Alor (sites of Makpan (triangles)and Tron Bon Lei (inverted triangles)).

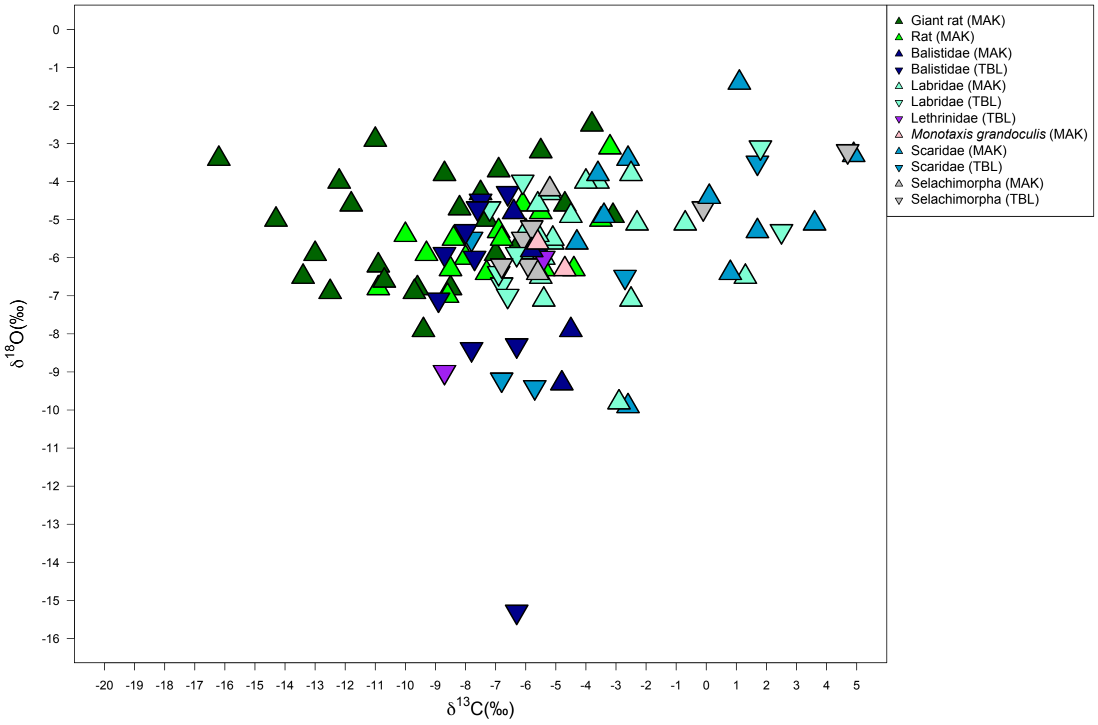

Supplementary Figure 14. Combined stable carbon ($\delta^{13}C$) and oxygen ($\delta^{18}O$) isotope data from terrestrial and marine faunal tooth enamel samples analyzed in this study from the island of Timor (sites of Asitau Kuru (circles) and Matja Kuru 2 (diamonds)).

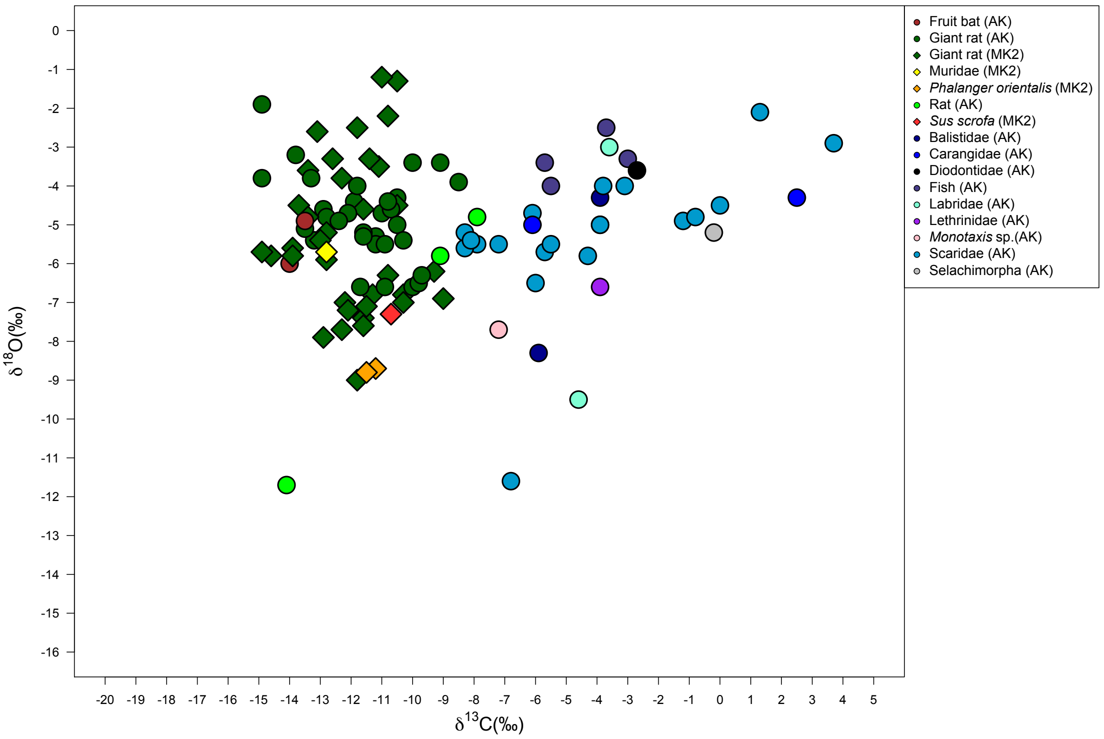

Supplementary Figure 15. Example FTIR plots of Modern (MOP3), Fossil Human (JH1), Fossil Terrestrial Fauna (JHF16), and Fossil Marine Fauna (FIFI1).

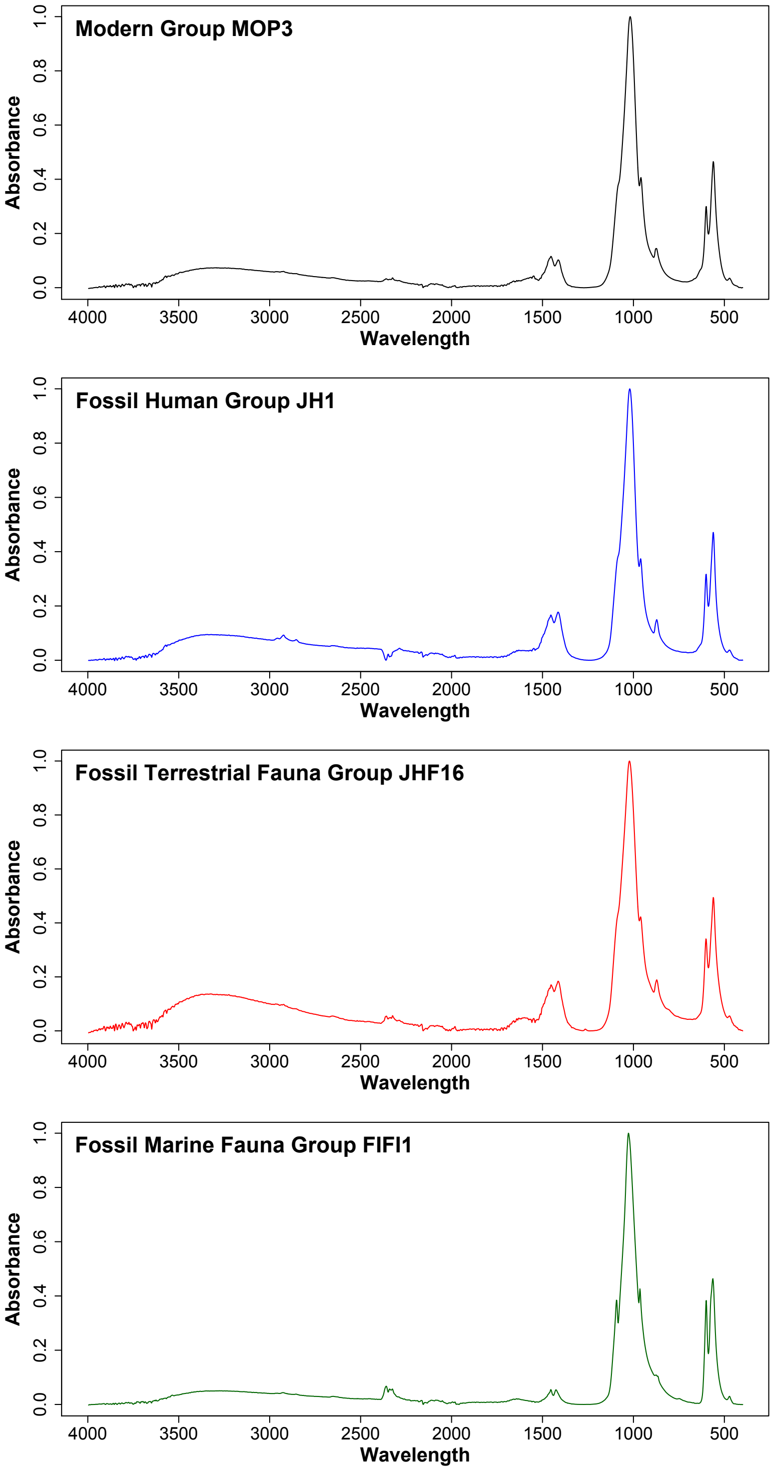

Supplementary Figure 16. Boxplots of A) API, B) BPI, C) WAMPI, D) PCI, and E) BAI for modern and fossil fauna. Each data point is the product of triplicate FTIR analyses. See Supplementary Table 13 for full list of samples. In all cases, boxplots show minimum, first quartile (25th percentile), median, third quartile (75th percentile), maximum, and outliers defined as being beyond 99.3% of the data probability density distribution [33]. Source data for Supplementary Figure 16 can be found in the accompanying Source Data file in tab 2 "Supplementary Figure 16".

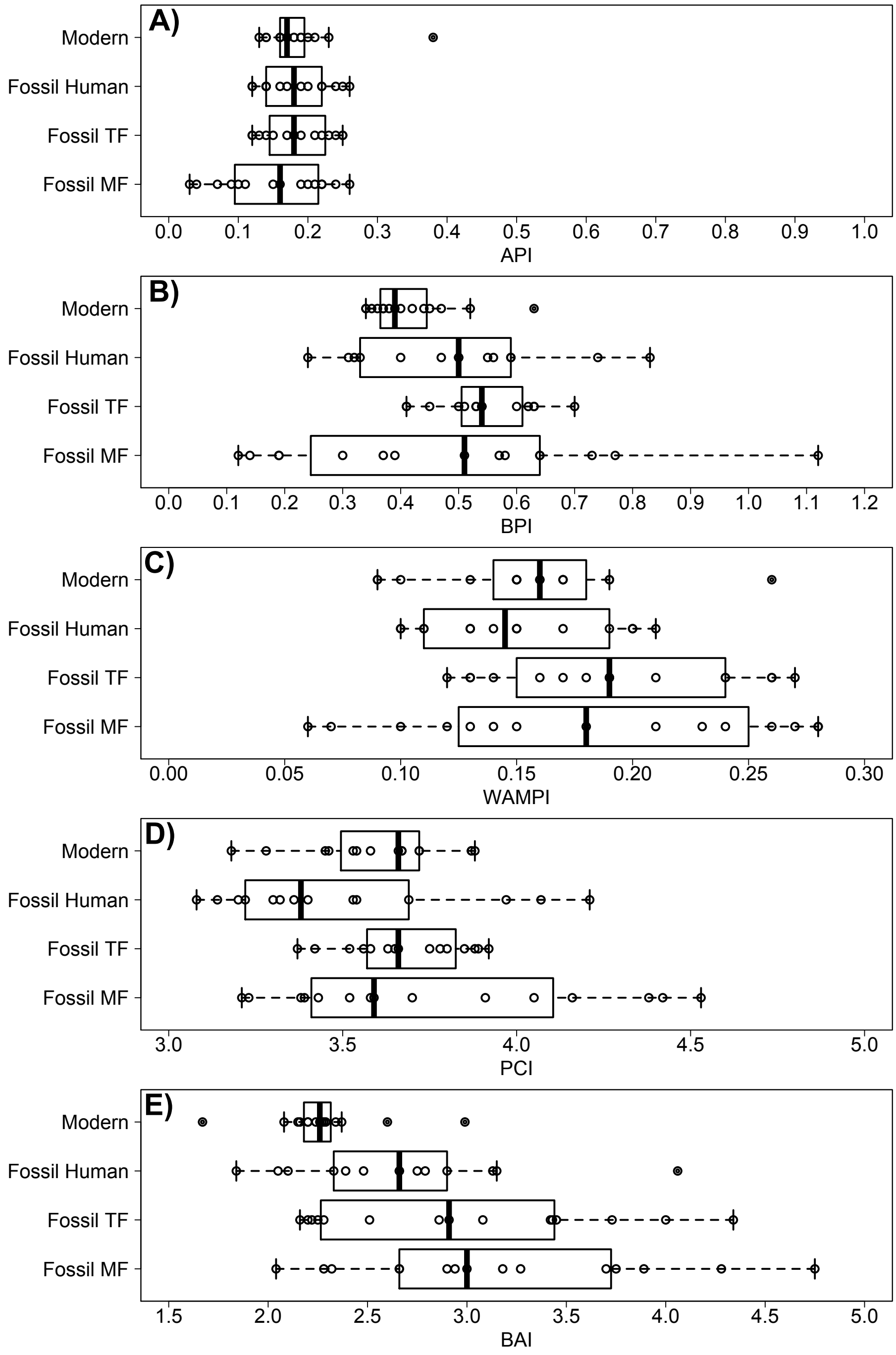

Supplementary Table 1. Chronometric ages available from Asitau Kuru (Squares B and C) and their stratigraphic relationship taken from O'Connor *et al.* [2], Langley *et al.* [4], and Shipton *et al.* [3]. All samples have been calibrated using the OxCal 4.3 software [34] and the IntCal13 calibration curve for charcoal or the Marine13 calibration curve for shell [1].

**Square B**				
**Spit**	**Material**	**Sample number**	**^{14}C**	**Calibrated date (cal. years BP) 2 sigma**
2				
3	Charcoal	Wk-19228	124 ± 32	10-273
4	*Turbo* sp.	Wk-19229	4962 ± 50	5454-5131
5				
6				
7				
9	*Trochus* sp.	Wk-19230	4580 ± 42	4900-4640
10				
11				
12				
13				
14				
15				
16	*Trochus* sp.	Wk-18157	4867 ± 42	5293-5032
18				
19				
20				
21	*Trochus* sp.	Wk-18158	5595 ± 43	6123-5887
22				
23	*Trochus* sp.	Wk-18159	5694 ± 45	6232-5972
24				
25				
26				
27				
28				
29				
30				
31				
32				
33	*Trochus* sp.	Wk-17832	5939 ± 45	6455-6265
34	*Nautilus* shell bead	Wk-19316	6118 ± 41	6654-6434
35				
37				
38				
39				
40	*Trochus* sp.	Wk-19231	8879 ± 78	9776-9393
41	*Olivia* shell bead	Wk-30500	6223 ± 26	6755-6586
42	*Olivia* shell bead	Wk-30501	5575 ± 27	6057-5887
43	*Olivia* shell bead	Wk-30502	13901 ± 45	16461-16057

46	*Olivia* shell bead	Wk-30503	9457 ± 32	10406-10207
49	*Haliotis* cf. *varia*	Wk-18160	14007 ± 146	17083-16151
50	*Haliotis* cf. *varia*	Wk-30504	13778 ± 43	16261-15907
56	*Olivia* shell bead; *Turbo* sp.	ANU-48106; Wk-19232	33294 ± 380; 35387 ± 534	38246-36136 41217-38853
66	*Trochus* sp.	Wk-17833	37267 ± 453	42440-41021
**Square C**				
1	Charcoal	S-ANU56520	Modern	
3	Charcoal	S-ANU56521	Modern	
4	Charcoal	S-ANU56523	107±27	145-15
6	Charcoal	S-ANU56524	3431±30	3732-3607
7	Charcoal	S-ANU56525	148±27	233-167
9	Charcoal	S-ANU56526	2514±30	2645-2491
10	Marine shell: *Tectus niloticus*	S-ANU56224	4421±28	4763-4451
12	Marine shell: *Tectus niloticus*	S-ANU56225	5198±30	5626-5471
14	Charcoal	S-ANU56527	3495±29	3847-3692
15	Charcoal	S-ANU56528	218±27	214-145
18	Marine shell: *Tectus niloticus*	S-ANU56226	5630±30	6144-5927
20	Marine shell: *Tectus niloticus*	S-ANU56227	5307±29	5733-5586
22	Marine shell: *Tectus niloticus*	S-ANU56230	5623±29	6128-5918
24	Marine shell: *Tectus niloticus*	S-ANU56231	5157±28	5581-5456
26	Marine shell: *Turbo argyrostomus*	S-ANU56232	5147±32	5580-5444
28	Marine shell: *Tectus niloticus*	S-ANU56233	6248±30	6785-6615
30	Marine shell: *Tectus niloticus*	S-ANU56235	5727±33	6248-6020
31	Charcoal	S-ANU56529	5270±32	5706-5570
31	Marine shell: *Tectus niloticus*	S-ANU56236	5467±31	5916-5741

33	Marine shell: *Tectus niloticus*	S-ANU56237	5443±29	5897-5731
37	Marine shell: *Tectus niloticus*	S-ANU56238	5447±29	5900-5733
42	Marine shell: *Turbo bruneus*	S-ANU56239	27938±181	31636-31065
43	Marine shell: *Tectus niloticus*	S-ANU56314	39473±749	44373-42001
46	Marine shell: *Turbo marmoratus*	S-ANU56316	34576±415	39770-37570
49	Marine shell: *Turbo marmoratus*	S-ANU56304	36873±539	42011-40036
53	Marine shell: *Tectus niloticus*	S-ANU56306	35376±449	40560-38583
54	Marine shell: *Turbo sestosus*	S-ANU56307	38403±651	43207-41336
56	Marine shell: *Turbo sestosus*	S-ANU56309	39111±695	43966-41803
57	Marine shell: *Tectus niloticus*	S-ANU56310	41572±939	46529-43085
58	Marine shell: *Conomurex luhuanus*	S-ANU56311	30168±268	34412-33455
59	Marine shell: *Haliatis sp.*	S-ANU56312	25544±164	29583-28780
60	Marine shell: *Conomurex luhuanus*	S-ANU56313	33766±384	38576-36532

Supplementary Table 2. Chronometric ages available from Lene Hara (Square F) and their stratigraphic relationship taken from Langley and O'Connor [4]. All samples have been calibrated using the OxCal 4.3 software [34] and the IntCal13 calibration curve for charcoal or the Marine13 calibration curve for shell [1].

Spit	Material	Sample number	14C	Calibrated date (cal. years BP) 2 sigma
7				
10	*Trochus niloticus*	ANU-12136	3305 ± 190	3603-2721
15				
16	*Trochus niloticus*	ANU-12041	3850 ± 70	4003-3607
17				
20	*Trochus niloticus*	ANU-12042	4370 ± 70	4775-4345
21				
22				
23	*Nautilus* shell bead; *Trochus niloticus*	OZG-893; ANU-12045	4900 ± 40<br>5270 ± 80	5313-5051<br>5841-5466
24				
25				
26				
27	*Nautilus* shell bead	NZA-16998	5782 ± 45	6300-6085
28				
29				
30				
31				
33				
35	?	OZG-894	6890 ± 50	7500-7295
36				
37				
40	*Oliva* sp. shell bead	NZA-16999	7945 ± 65	8556-8283
41	*Oliva* sp. shell bead	OZG-895	7830 ± 50	9575-7378
42	*Trochus niloticus* fish hook	NZA-17000	9741 ± 60	10841-10491
43	*Tridacna maxima*	ANU-12040	10050 ± 80	11209-10791

Supplementary Table 3. Chronometric ages available from Matja Kuru 1 (Square AA) and 2 (Squares B, C, and D) and their stratigraphic relationship taken from Langley and O'Connor [4]. All samples have been calibrated using the OxCal 4.3 software [34] and the IntCal13 calibration curve for charcoal or the Marine13 calibration curve for shell [1].

**Matja Kuru 1 Square A**				
**Spit**	**Material**	**Sample number**	**14C**	**Calibrated date (cal. years BP) 2 sigma**
5	*Lambis lambis*	ANU-11835	4650 ± 70	5115-4680
8	*Lambis lambis*	NZA-16135	5005 ± 40	5456-5274
12	*Acanthopleura* sp.	NZA17007	3776 ± 40	3832-3595
14	*Cymbiola vespertilio*	ANU-11632	3840 ± 70	3985-3597
25	*Trochus* sp.	OZF782	5720 ± 50	
31	*Tectus niloticus*	ANU11623	5680 ± 110	
35	*Marine shell*	0ZF783	4300 ± 50	
**Matja Kuru 2 Square B**				
4	Mytilidae sp.	Wk-41348	2515 ± 20	2285-2111
7	Mytilida. *Perna viridis* sp.	Wk-41349	2539 ± 20	2296-2136
12A	*Mytilidae* sp.	Wk-41351	2514 ± 20	2285-2110
13A	*Mytilidae* sp.	Wk-41352	2529 ± 20	2291-2127
16	*Nautilus* sp.	Wk-41353	9414 ± 23	10335-10180
18	*Nautilus* sp.	Wk-41354	9479 ± 26	10420-10225
23	*Nautilus* sp.	Wk-41355	9018 ± 22	9815-9561
28	*Acanthopleura* sp.	Wk-41356	11330 ± 29	12904-12676
31	*Nautilus* sp.	Wk-41357	9106 ± 22	9955-9690
35	*Nautilus* sp.	Wk-41358	9006 ± 21	9780-9545
39	*Nautilus* sp.	Wk-41359	9110 ± 23	9998-9696
42	*Nautilus* sp.	Wk-41122	9391 ± 20	10276-10166
46	*Nautilus* sp	Wk-41360	9231 ± 25	10155-9923
51	*Tectus sp.*	Wk-41361	31094 ± 268	35143-34102

60	*Tectus fenestrata*	Wk-41362	31,070 ± 271	35120-34083
**Matja Kuru 2 Square C**				
C44	*Nautilus* sp.	Wk-41363	9473 ± 22	10405-10226
**Matja Kuru 2 Square D**				
**Spit**	**Material**	**Sample number**	**14C**	**Calibrated date (cal. years BP) 2 sigma**
10	*Modulus philippinosus*	NZA-16136	2450 ± 40	2255-1978
13	*Celtis* sp.; Marine shell	OZG-537; OZG-538	2510 ± 50; 3190 ± 40	2747-2427; 3492-3341
15	*Nautilus* shell bead	NZA-18656	8966 ± 55	9817-9499
16				
17	*Acanthopleura* sp.	NZA-17008	10292 ± 60	11609-11146
22				
24	Drilled *Tectus* shell bead	OZG-896	4490 ± 40	4808-4554 May be displaced to disturbance from dog burial in spit 25
25	*Chiton* sp.	NZA-17009	10078 ± 60	11215-10884
26	*Chiton* sp.	NZA-16137	9650 ± 55	10691-10381
27				
29				
31	*Nautilus* shell bead	OZG-899	9190 ± 50	10155-9801
32	*Nautilus* shell bead; *Oliva* shell bead	NZA-17001; OZG-897	9205 ± 55<br>9260 ± 60	10172-981310215-9860
35	*Nautilus* shell bead	OZG-898	9260 ± 50	10200-9909
36	*Celtis* seed	OZG 737	26690 ± 170	31135-30640
41	*Nerita albicilla*	NZA 16177	31060 ± 310	35201-34026
44	*Turbo cinereus*	NZA 16178	31660 ± 320	35882-34575
47	Marine shellfish	OZF 785	32220 ± 300	36325-35046
52 (2014)	*Tectus niloticus*	Wk-41364	34,650 ± 418	39844-37721

Supplementary Table 4. Chronometric ages available from Makpan main pit (Square B) and their stratigraphic relationship. All samples have been calibrated using the OxCal 4.3 software [34] and the IntCal13 calibration curve for charcoal or the Marine13 calibration curve for shell [1].

**Spit**	**Layer**	**Material**	**Sample number**	**14C**	**Calibrated date (cal. years BP) 2 sigma**
1	2	Charcoal	ANU-51814	714±30	565 - 700
2	2	Charcoal	ANU-52311	1018±24	835 - 974
3	2	Charcoal	ANU-51816	1214±28	1,016 - 1,255
6	4	Charcoal	ANU-52312	2287±26	2,183 - 2,352
9	4	Charcoal	ANU-52313	3087±25	3,230 - 3,367
10	5	Charcoal	ANU-52314	6775±29	7,582 - 7,670
11	5	*Tooth enamel – *Homo sapiens*	ANU-56516	7041±36	7,795 - 7,950
13	5	*Charcoal near skull	ANU-53911	7936±32	8,639 - 8,978
13	5	*Turbo* sp.	ANU-51606	7867±33	8,241 - 8,405
16	6	Charcoal	ANU-52316	7945±30	8,645 - 8,979
20	7	Muricidae	ANU-53622	9298±41	9,958 - 10,228
21	10	Charcoal	ANU-52317	10050±34	11,365 - 11,759
25**	10	Charcoal	ANU-52318	10073±34	11,400 - 11,804
29	10	Charocal	ANU-52325	10055±34	11,393 - 11,764
30	10	Charcoal	ANU-51410	10100±38	11,405 - 11,970
31	10	Charcoal	ANU-52321	10118±38	11,411 - 12,003
32	10	Charcoal	ANU-52326	10162±35	11,650 - 12,027
33	13	Charcoal	ANU-52320	10087±34	11,402 - 11,928
35	15	*Tridacna* sp.	ANU-53621	10483±45	11,355 - 11,900
37	17	Muricidae	ANU-53620	11842±52	13,191 - 13,434
38	17	Charcoal	ANU-52327	10552±37	12,420 - 12,635
42	17	*Haliotis* sp.	ANU-51610	10903±45	12,234 - 12,599
45	17	Charcoal	ANU-52330	11609±37	13,346 - 13,557
47	17	*Patella* sp.	ANU-51416	11003±42	12,458 - 12,662
48	17	Charcoal	ANU-52331	10567±35	12,423 - 12,654
49	17	*Patella* sp.	ANU-51612	12237±43	13,529 - 13,842
52	17	Muricidae	ANU-53617	11649±48	12,987 - 13,279
53	17	Muricidae	ANU-53616	11821±48	13,177 - 13,411
54	17	Muricidae	ANU-53614	13138±50	14,971 - 15,340
57	17	Muricidae	ANU-53613	11783±48	13,144 - 13,375
61	18	Charcoal	ANU-51412	17893±69	21,445 - 21,895
62	18	*Patella* sp.	ANU-51417	35130±414	38,454 - 40,208
67	18	*Asaphis violascens*	ANU-53610	19894±87	23,130 - 23,758
68	18	*Turbo* sp.	ANU-53609	35232±427	38,508 - 40,342

**Indicates samples recovered from Square D, included based on their association with the human remains sampled here. **Radiocarbon samples from spits 25 and above were collected from the north wall of the excavation which incorporates Squares A and B. All samples from spit 29 down are from Square B only.*

Supplementary Table 5. Chronometric ages available from Tron Bon Lei (Test Pit B) and their stratigraphic relationship taken from Samper Carro *et al.* [22, 24, 35] and O'Connor *et al.* [26]. All samples have been calibrated using the OxCal 4.3 software [34] and the IntCal13 calibration curve for charcoal or the Marine13 calibration curve for shell [1].

Spit	Material	Sample number	14C	Calibrated date (cal. years BP)
1	*Turbo* sp.	ANU-42723	3275 ± 20	3193-3011
3	*Barbatia* sp.	ANU-42724	3395 ± 20	3343-3186
4	Charcoal	ANU-41918	3065 ± 20	3350-3215
5	Oyster	ANU-42725	5010 ± 20	5436-5296
6	Charcoal	ANU-41919	3325 ± 20	3613-3480
7	Charcoal	ANU-41920	3210 ± 20	3462-3380
8	Charcoal	ANU-41921	3390 ± 20	3692-3580
SW-6-L6	Charcoal	ANU-40123	6620 ± 30	7570-7441
SW-5-L8	Charcoal	ANU-40121	7060 ± 30	7955-7839
SW-4-L8	Charcoal	ANU-40120	7205 ± 30	8153-7955
NW-3-L9	Charcoal	ANU-40039	7250 ± 25	8160-8005
NW-2-L9	Charcoal	ANU-39539	7355 ± 35	8306-8036
NW-1-L10	Charcoal	ANU-39538	8745 ± 35	9891-9564
SW-2-L10	Charcoal	ANU-40118	8955 ± 40	10225-9919
SW-R2-L11	*Haliotis asinina*	ANU-40128	9340 ± 35	10244-10112
40	Charcoal	ANU-40125	10140 ± 45	12034-11502
41	Trochus sp. fishook	ANU-41825	10230 ± 30	11300-11133
41	Charcoal	ANU-40124	10445 ± 50	12543-12117
SW-P-L12	*Haliotis asinina*	ANU-41029	16210 ± 60	19235-18893
58	*Nerita* sp.	ANU-40130	17630 ± 70	21000-20560

The samples with a SW suffix were collected in situ directly from the section and are assigned to Layers rather than spits. Their location and relationship to other samples can be seen on the section in Supplementary Figures 10-12.

Supplementary Table 6. Overall Phasing used for the island of Timor in this study and its stratigraphic correlation with contexts from the different sites. Phase letters used as shorthand in the statistical analyses are also shown (see also Supplementary Data 1 and 2).

Regional Phase Used in Study	Contexts Incorporated from Different Sites Sampled
Neolithic/ Late Holocene ("D"): 4,000-0 years ago	Asitau Kuru Square B 1-14, Asitau Kuru Square C 1-16, Matja Kuru 2 Squares B and D Spits 1-13
Early to Middle Holocene ("C"): 11,000-4001 years ago	Asitau Kuru Square B 15-42, Asitau Kuru Square C 17-41, Lene Hara Square F Spit 37-43, Matja Kuru 2 16-46, Matja Kuru Square D 15-35, Matja Kuru 1 Square AA Spits 7-20
Terminal Pleistocene ("B"): 20,000-11,001 years ago	Asitau Kuru Square B 43-52, Asitau Kuru Square C 42-50, Matja Kuru 1 Square AA Spits 21-25
Late Pleistocene pre-LGM ("A"): 46,000-29,000 years ago	Asitau Kuru Square B 53-67, Asitau Kuru Square C 51-60, Matja Kuru 2 Square B Spits 47-78, Matja Kuru 2 Square D 36-42

Supplementary Table 7. Overall Phasing used for the island of Alor in this study and its stratigraphic correlation with contexts from the different sites. Phase letters used as shorthand in the statistical analyses are also shown (see also Supplementary Data 1 and 2).

Regional Phase Used in Study	Contexts Incorporated from Different Sites Sampled
Neolithic/ Late Holocene ("C"): 4,000-0 years ago	Makpan Squares A, B, C, and D Spits 1-8, Tron Bon Lei All Squares Layers 1-5
Terminal Pleistocene to Middle Holocene ("B"): 15,000-7,400 years ago	Makpan Squares A, B, C, and D 9-23, Makpan Square B Spits 24-57, Tron Bon Lei All Squares Layers 6-11
Late Pleistocene pre-LGM ("A"): 40,000-21,000 years ago	Makpan Square B Spits 58-68

Supplementary Table 8. Kruskal Wallis Wilcoxon pairwise (two-sided) comparison using holm adjustment method table of $\delta^{18}O$ by Phase for data from Timor showing p-values to three decimal places. For correspondence of Phases to date ranges see Supplementary Table 6. Bold and "*" show where the p=value is less than 0.05.

**Phase**	**A**	**B**	**C**
**B**	1.000	-	-
**C**	0.071	0.056	-
**D**	0.082	0.055	1.000

Supplementary Table 9. Kruskal Wallis Wilcoxon pairwise (two-sided) comparison using holm adjustment method table of $\delta^{13}C$ by Phase for data from Alor showing p-values to three decimal places. For correspondence of Phases to date ranges see Supplementary Table 7. Bold and "*" show where the p=value is less than 0.05.

**Phase**	**A**	**B**
**B**	0.760	-
**C**	**0.015***	**0.002***

Supplementary Table 10. Kruskal Wallis Wilcoxon pairwise (two-sided) comparison table of $\delta^{13}C$ by taxa for all compiled marine faunal data showing p-values to three decimal places. * = p=<0.05.

**Taxa**	**Balistidae**	**Carangidae**	**Diodontidae**	**Fish**	**Labirdae**	**Lethrinidae**	**Monotaxis sp.**	**Scaridae**
**Carangidae**	1.000	-	-	-	-	-	-	-
**Diodontidae**	1.000	1.000	-	-	-	-	-	-
**Fish**	0.598	1.000	1.000	-	-	-	-	-
**Labridae**	**0.019***	1.000	1.000	1.000	-	-	-	-
**Lethrinidae**	1.000	1.000	1.000	1.000	1.000	-	-	-
**Monoaxis sp.**	1.000	1.000	1.000	1.000	1.000	1.000	-	-
**Scaridae**	**0.025***	1.000	1.000	1.000	1.000	1.000	1.000	-
**Selachimorpha**	0.626	1.000	1.000	1.000	1.000	1.000	1.000	1.000

Supplementary Table 11. Results of post-Hoc Tukey Honest Significant Differences pairwise multiple comparison with for BAI of 'Modern' human and animal samples, 'Fossil Human' samples, 'Fossil Terrestrial Fauna (TF)' samples, and 'Fossil Marine Fauna (MF)' samples. 95% confidence interval of difference is indicated alongside 95% probability of lower and upper bounds of this difference, and p-values shown to three decimal places. Bold and "*" show where the p=value is less than 0.05. This test and function is applied to a fitted one-way ANOVA and incorporates an adjustment for sample size that provides sensible intervals for mildly unbalanced designs [33].

**Group**	**Difference**	**Lower**	**Upper**	**Probability adjacent**
Fossil MF-Fossil Human	0.511	-0.093	1.115	0.125
Fossil TF-Fossil Human	0.326	-0.278	0.930	0.487
Modern-Fossil Human	-0.390	-0.994	0.214	0.327
Fossil TF-Fossil MF	-0.185	-0.779	0.408	0.841
Modern-Fossil MF	-0.901	-1.495	-0.308	**0.001***
Modern-Fossil TF	-0.716	-1.309	-0.123	**0.012***

Supplementary Table 12. Description of the FTIR indices of enamel bioapatite. B and V denote the height of the bands and the valleys, respectively. Numbers in parantheses represent the approximate positions of the apparent bands (adapted from [36]).

**Indices**	**Formulas**	**References**
PCI (Phosphate Crystallinity Index)<br>other names:<br>CI_{IR} (Crystallinity Index $_{InfraRed}$)<br>IRSF (InfraRed Splitting Factor)	$\frac{B(605)+B(565)}{V(590)}$	37, 38, 39
BPI (B-carbonate on Phosphate Index)	$\frac{B(1415)}{B(605)}$	40
API (A-carbonate on Phosphate Index)	$\frac{B(1545)}{B(605)}$	36
BAI (relative amount of B- to A-site carbonate)	$\frac{B(1415)}{B1540}$	36
WAMPI (Water-Amide on Phosphate Index)	$\frac{B(1650)}{B(605)}$	36

References

1. Reimer, P.J. *et al.* IntCal13 and Marine13 Radiocarbon Age Calibration Curves 0-50,000 Years cal BP. *Radiocarbon* **55**, 1869-1887 (2013).

2. O'Connor, S., Ono, R. & Clarkson, C. Pelagic fishing at 42,000 years before the present and the maritime skills of modern humans. *Science* **334**, 1117-1121 (2011).

3. Shipton, C. *et al.* A new 44,000-year sequence from Asitau Kuru (Jerimalai), Timor-Leste, indicates long-term continuity in human behaviour. *Archaeol Anthropol Sci* **11**, 5717-5741 (2019).

4. Langley, M.C. & O'Connor, S. An enduring shell artefact tradition from Timor-Leste: *Oliva* bead production from the Pleistocene to Late Holocene at Jerimalai, Lene Hara, and Matja Kuru 1 and 2. *PLoS ONE* **11**, e0161071 (2016).

5. Langley, M.C., O'Connor, S. & Piotto, E. 42,000-year-old worked and pigment-stained *Nautilus* shell from Jerimalai (Timor-Leste): Evidence for an early coastal adasptation in ISEA. *J Hum Evol* **97**, 1-16 (2016).

6. O'Connor, S. & Aplin, K. A matter of balance: An overview of Pleistocene occupation history and the impact of the Last Glacial Phase in East Timor and the Aru Islands, eastern Indonesia. *Archaeol Ocean* **42**, 82-90 (2007).

7. Glover, I. *Archaeology in Eastern Timor, 1966–67.* Terra Australis 11 (Department of Prehistory, Research School of Pacific Studies, Australian National University, Canberra, 1986).

8. Meijer, H.J., Louys, J. & O'Connor, S. First record of avian extinctions from the Late Pleistocene and Holocene of Timor Leste. *Quat Sci Rev* **203**, 170-184 (2019).

9. O'Connor, S. & Ono, R. The case for complex fishing technologies: a response to Anderson. *Antiquity* **87**, 885-888 (2013).

10. Anderson, A. The antiquity of sustained offshore fishing. *Antiquity* **87**, 879-895 (2013).

11. O'Connor, S., Spriggs, M. & Veth, P. Direct dating of shell beads from Lene Hara Cave, East Timor. *Aust Archaeol* **55**, 18-21 (2002).

12. O'Connor, S., Spriggs, M. & Veth, P. Excavation at Lene Hara Cave establishes occupation in East Timor at least 30,000-35,000 years ago. *Antiquity* **76**, 45-49 (2002).

13. O'Connor, S. *et al.* Faces of the ancestors revealed: Discovery and dating of a Pleistocene-age petroglyph in Lene Hara Cave, East Timor. *Antiquity* **84**, 649-665 (2010).

14. O'Connor, S. & Veth, P. Early Holocene shell fish hooks from Lene Hara Cave, East Timor establish complex fishing technology was in use in Island South East Asia five thousand years before Austronesian settlement. *Antiquity* **79**, 249-256 (2005).

15. Spriggs, M., O'Connor, S. & Veth, P.M. Vestiges of early pre-agricultural economy in the landscape of East Timor - recent research. In *Fishbones and Glittering Emblems: Proceedings from the EurASEEA Sigtuna Conference* (eds. A. Kallen, A. Karlstrom), 49-58 (Museum of Far Eastern Antiquities, Stockholm, 2003).

16. O'Connor, S., Robertson, G. & Aplin, K.P. Are osseous artefacts a window to perishable material culture? Implications of an unusually complex bone tool from the Late Pleistocene of East Timor. *J Hum Evol* **67**, 108-119 (2014).

17. O'Connor, S. 2015 Rethinking the Neolithic in Island Southeast Asia, with particular reference to the archaeology of Timor-Leste and Sulawesi. *Archipel Études interdisciplinaires sur le monde insulindien* **90**, 15-48 (2015).

18. Gonzalez, A. *et al.* A. 3000 year old dog burial in Timor-Leste. *Aust Archaeol* **76**, 13–20 (2013).

19. Koesoemadinata, S. & Noya, N. *Peta Geologi Lembar Lomblen, Nusatenggara Timur* (Pusat Penelitian dan Pengembangan Geologi, Bandung, Indonesia, 1989).

20. Louys, J. *et al.* New genus and species of giant rat from Alor Island, Indonesia. *J Asia-Pacific Biodiv* **11**, 503-510 (2018).

21. Hawkins, S. *et al.* Human palaeoecological interactions and owl roosting at Tron Bon Lei, Alor Island, eastern Indonesia. *JICA* **13**, 371-387 (2018).

22. Samper Carro, S.C., Louys, J. & O'Connor, S. Methodological considerations for icthyoarchaeology from the Tron Bon Lei sequence, Alor, Indonesia. *Archaeol Res Asia* **12**, 11-22 (2017).

23. Samper Carro, S.C. *et al.* Human maritime subsistence strategies in the Lesser Sunda Islands during the terminal Pleistocene-early Holocene: New evidence from Alor, Indonesia. *Quat Int* **416**, 64-79 (2016).

24. Samper Carro, S.C. *et al.* Somewhere beyond the sea: Human cranial remains from the Lesser Sunda Islands (Alor Island, Indonesia) provide insights on Late Pleistocene peopling of Island Southeast Asia. *J Hum Evol* **134**, 102638 (2019).

25. O'Connor, S. *et al.* Hominin dispersal and settlement east of Huxley's Line: The role of sea-level changes, island size, and subsistence behaviour. *Curr Anthropol* **58**, S567-S582 (2017).

26. O'Connor, S. *et al.* Fishing in life and death: Pleistocene fish-hooks from a burial context on Alor Island, Indonesia. *Antiquity* **91**, 1451-1468 (2017).

27. O'Connor, S. *et al.* Kisar and the archaeology of small islands in the Wallacean Archipelago. *JICA **14***, 198-225 (2019).

28. Reepmeyer, C. *et al.* Kisar, a small island participant in an extensive maritime obsidian network in the Wallacean Archipelago. *Archaeol Res Asia* **19**, 100139 (2019).

29. Shipton, C. *et al.* Shell Adzes, exotic obsidian, and inter-island voyaging in the Early and Middle Holocene of Wallacea. *JICA*
DOI: 10.1080/15564894.2019.1581306 (2019).

30. Hawkins, S. *et al.* Oldest human occupation of Wallacea at Laili Cave, Timor-Leste, shows broad-spectrum foraging responses to late Pleistocene environments. *Quat Sci Rev* **171**, 58-72 (2017).

31. Monk, K.A., de Fretes, Y. & Reksodiharjo-Lilley, G. *The Ecology of Nusa Tenggara and Maluku* (Periplus Editions, Jakarta, Indonesia, 1997).

32. Gagan, M.K. *et al.* 2004. Post-glacial evolution of the Indo-Pacific warm pool and El Nino-Southern Oscillation. *Quat Int* **118**, 127-143 (2004).

33. R Core Team. R: A language and environment for statistical computing. R Foundation for Statistical Computing, Vienna, Austria (2013).

34. Bronk Ramsey, C. Bayesian analysis of radiocarbon dates. *Radiocarbon* **51**, 337-360 (2009).

35. Samper Carro, S.C., Louys, J., O'Connor, S. Shape does matter: A geometric morphometric approach to shape variation in Indo-Pacific fish vertebrae for habitat identificastion. *J Archaeol Sci* **99**, 124-134 (2018).

36. Roche, D. *et al.* Preservation assessment of Miocene-Pliocene tooth enamel from Tugen Hills (Kenyan Rift Valley) - through FTIR, chemical and stable-isotope analyses. *J Archaeol Sci* **37**, 1690-1699 (2010).

37. Sponheimer, M. & Lee-Thorp, J.A. Isotopic evidence for the diet of an early hominid, *Australopithecus africanus*. *Science* **283**, 368-370 (1999).

38. Shemesh, A. Crystallinity and diagenesis of sedminearty apatite. *Geochim Cosmochim Acta* **54**, 2433-2438 (1980).

39. Weiner, S. & Bar-Yosef, O. States of preservation of bones from prehistoric sites in the Near East: a survey. *J Arch Sci* **17**, 187-196 (1990).

40. LeGeros, R.Z. Calcium phosphates in Oral Biology and Medicine. *Monogr Oral Sci* **15**, 1-201 (1991).
