# Supplementary material for: Isotopic evidence for initial coastal colonization and subsequent diversification in the human occupation of Wallacea
Source: Nat Commun. 2020 Apr 29;11:2068. doi: 10.1038/s41467-020-15969-4 (PMC7190613; doi:10.1038/s41467-020-15969-4)
Supplement: Supplementary file 3 — Reporting Summary [file 41467_2020_15969_MOESM3_ESM.pdf]

nature research

Corresponding author(s): Patrick Robetrs, Sue O'Connor

Last updated by author(s): Mar 29, 2020

Reporting Summary

Nature Research wishes to improve the reproducibility of the work that we publish. This form provides structure for consistency and transparency in reporting. For further information on Nature Research policies, seeAuthors & Referees and theEditorial Policy Checklist .

Statistics

For all statistical analyses, confirm that the following items are present in the figure legend, table legend, main text, or Methods section.

n/a	Confirmed	
☐	☒	The exact sample size (*n*) for each experimental group/condition, given as a discrete number and unit of measurement
☐	☒	A statement on whether measurements were taken from distinct samples or whether the same sample was measured repeatedly
☐	☒	The statistical test(s) used AND whether they are one- or two-sided<br>*Only common tests should be described solely by name; describe more complex techniques in the Methods section.*
☐	☒	A description of all covariates tested
☐	☒	A description of any assumptions or corrections, such as tests of normality and adjustment for multiple comparisons
☐	☒	A full description of the statistical parameters including central tendency (e.g. means) or other basic estimates (e.g. regression coefficient) AND variation (e.g. standard deviation) or associated estimates of uncertainty (e.g. confidence intervals)
☐	☒	For null hypothesis testing, the test statistic (e.g. *F*, *t*, *r*) with confidence intervals, effect sizes, degrees of freedom and *P* value noted<br>*Give P values as exact values whenever suitable.*
☒	☐	For Bayesian analysis, information on the choice of priors and Markov chain Monte Carlo settings
☒	☐	For hierarchical and complex designs, identification of the appropriate level for tests and full reporting of outcomes
☒	☐	Estimates of effect sizes (e.g. Cohen's *d*, Pearson's *r*), indicating how they were calculated

Our web collection on statistics for biologists contains articles on many of the points above.

Software and code

Policy information about availability of computer code

Data collection	Isodat 3.0 software from Thermo Electron Corporation
Data analysis	R Core Team, 2013 - Free Program R Software, OPUS 8.5 FTIR software from Bruker, and OxCal 4.3

For manuscripts utilizing custom algorithms or software that are central to the research but not yet described in published literature, software must be made available to editors/reviewers. We strongly encourage code deposition in a community repository (e.g. GitHub). See the Nature Research guidelines for submitting code & software for further information.

Data

Policy information about availability of data

All manuscripts must include a data availability statement. This statement should provide the following information, where applicable:

- Accession codes, unique identifiers, or web links for publicly available datasets
- A list of figures that have associated raw data
- A description of any restrictions on data availability

All of the data reported in the paper are presented in the main text or in the Supplementary Notes, Tables, Figures, and Data files. The source data underlying Figure 2 and Supplementary Figure 16 are provided as a Source Data file. The source data for Figure 2, as well as Supplementary Data Files 1 and 2, also underlie Figures 3-6. All data supporting the findings of this study are available in existing publications and the Supplementary Information provided alongside this manuscript . The faunal and human remains sampled from Timor are curated at the School of Asia and the Pacific, Australian National University, Australia under the site codes J (Asitau Kuru), MK (Matja Kuru), and LH (Lene Hara). These materials are to be returned to Timor-Leste, Indonesia upon construction of a national museum storehouse. The faunal and human remains from Alor are housed in the Departemen Arkeologi, Universitas Gadjah Mada, Indonesia, under the site codes TBL (Tron Bon Lei) and MP (Makpan). All site codes are followed by suffixes of a single letter denoting the excavation square and a number denoting the spit.

Field-specific reporting

Please select the one below that is the best fit for your research. If you are not sure, read the appropriate sections before making your selection.

☐ Life sciences ☐ Behavioural & social sciences ☒ Ecological, evolutionary & environmental sciences

For a reference copy of the document with all sections, see nature.com/documents/nr-reporting-summary-flat.pdf

Ecological, evolutionary & environmental sciences study design

All studies must disclose on these points even when the disclosure is negative.

Study description

We test the ecological adaptations of the earliest members of our own species in Wallacea by means of isotopic analysis of archaeological human and animal tooth enamel from two islands (Timor and Alor). We apply stable carbon (δ13C) and oxygen (δ18O) isotope analyses to human and faunal tooth enamel from six Late Pleistocene/Holocene archaeological sequences on the islands of Timor and Alor in order to determine the varying reliance of early colonisers of Wallacea on tropical forest and terrestrial versus marine resources. Ample terrestrial and marine animal remains also allow us to build the first detailed palaeoenvironmental records for Pleistocene Wallacea and test assumptions in relation to: 1) pure C3 terrestrial environments on Timor and Alor in the past; 2) the δ13C distinction between available terrestrial and marine resources and; 3) environmental shifts across the Pleistocene-Holocene boundary proposed elsewhere in Southeast Asia. The preservation of the analyzed tooth enamel samples was also checked using Fourier Transform Infrared Spectroscopy (FTIR) as per previous work.

Research sample

We sampled all of the available identified human and animal teeth from the Late Pleistocene-Holocene deposits of Asitau Kuru, Matja Kuru 1 and 2, Lene Hara on the island of Timor and Makpan, and Tron Bon Lei on the island of Alor. Sample size was determined based on availability and no sample size calculation was performed.

Existing, including published, stratigraphic and chronological information was compiled in order to enable the placement of the resulting samples in their proper context. All of the available dates used, stratigraphic information, and accompanying archaeological finds can be found in Supplementary Note 1, Supplementary Figures 1-12, and Supplementary Tables 1-7. The available samples were selected on the basis of occupation 'Phases' noted and published for each archaeological site (Supplementary Note 1, Supplementary Tables 1-7). Based on these established sequences, samples were also grouped into an overall 'Phasing' for each of the islands of Timor and Alor to provide larger sample sizes for the broader evaluation of adaptive context for the arrival of humans on each island, hypothesized palaeoenvironmental changes across the Terminal Pleistocene/Holocene boundary, and changes following the arrival of 'Neolithic' material culture during the Holocene (Supplementary Note 1, Supplementary Data 1 and 2).

14 fossil human, 15 fossil terrestrial fauna, and 15 marine fauna samples were subjected to FTIR analysis following pre-treatment in order to determine the potential for diagenetic structural and compositional modification of enamel after pretreatment (Supplementary Data 3). Samples were randomly subjected to cover a variety of the temporal 'Phases' and all of the sites studied (Supplementary Data 3). The fossil spectra were compared to those available for 15 modern primate and cervid samples and historical (late 19th and early 20th century) human enamel samples from populations living in tropical forest environments in Sri Lanka already published by Roberts et al. (Supplementary Data 3).

Sampling strategy

We sampled all of the available identified human and animal teeth from the Late Pleistocene-Holocene deposits of Asitau Kuru, Matja Kuru 1 and 2, Lene Hara on the island of Timor and Makpan, and Tron Bon Lei on the island of Alor. Sample size was determined based on availability and no sample size calculation was performed.

Tooth identification and analyses were conducted at the Australian National University (ANU). Fish, reptile, and non-murid mammal identifications were facilitated through comparisons with specimens from the ANU Archaeology and Natural History Osteology Laboratory reference collection. Murid identifications were facilitated through comparisons with archaeological and fossil specimens collected from previous ANU expeditions, material held in KA's private collections, informed by the Australian National Wildlife Collection of the Commonwealth Scientific and Industrial Research Organization (CSIRO) National Facilities and Collections, and descriptions and illustrations in Aplin and Helgen and Glover.

All teeth or teeth fragments were cleaned using air-abrasion to remove any adhering external material. Enamel powder for bulk analysis was obtained using gentle abrasion with a diamond-tipped drill along the full length of the buccal surface in order to ensure a representative measurement for the entire period of enamel formation. All enamel powder was pretreated to remove organic or secondary carbonate contaminates. This method followed established protocols that have been applied elsewhere to Pleistocene tooth enamel in the tropics, where it has proven to be effective, enabling future comparison between datasets. Samples were washed in 1.5% sodium hypochlorite for 60 minutes, followed by three rinses in purified H2O and centrifuging, before 0.1M acetic acid was added for 10 minutes, followed by another three rinses in purified H2O. Samples were then lysophilized for 24 hours.

Data collection

Following reaction with 100% phosphoric acid, gases evolved from the samples were measured by stable carbon and oxygen isotope analysis using a Thermo Gas Bench 2 connected to a Thermo Delta V Advantage Mass Spectrometer at MPI-SHH. δ13C and δ18O values were compared against International Standards (IAEA-603 (δ13C = 2.5; δ18O = -2.4); IAEA-CO-8 (δ13C = -5.8; δ18O = -22.7); USGS44 (δ13C = -42.2)) and in-house standard (MERCK (δ13C = -41.3; δ18O = -14.4)) using Isodat 3.0 software from Thermo Electron Corporation. JZ, SM, BF, and PR performed and supervised data collection. Replicate analysis of MERCK standards suggests that machine measurement error is c. ± 0.1‰ for δ13C and ± 0.2‰ for δ18O. Overall measurement precision was studied through the measurement of repeat extracts from a bovid tooth enamel standard (n = 20, ± 0.2‰ for δ13C and ± 0.3‰).

For FTIR we used the empirical indices from Sponheimer and Lee-Thorp, and Roche et al. (see main text references) to characterize the crystal-chemical properties of enamel bioapatite (Supplementary Table 10). The possible presence of calcite was assessed in all samples by checking for a peak at 711 cm-1. For all samples, powdered enamel was analysed between 400 and 4,000 cm-1 by Fourier Transform Infrared spectroscopy with Attenuated Total Reflectance (FTIR-ATR – Bruker Vertex 70v) using the OPUS 8.5 software from Bruker. Each sample was measured three times. The background was subtracted and a baseline correction was carried out using OPUS 8.5 software from Bruker. The baselines of the spectra were normalised and all three spectra of each sample were averaged before calculation of the various infrared indices. To ensure better reproducibility of the measurements, only spectra with a minimum absorbance of 0.06 for the highest phosphate band at ~1035 cm-1 were taken into account. The reproducibilities of the indices BPI, API, BAI, and PCI are ±0.01, ±0.004, ±0.1 and ±0.1, respectively. PR performed and supervised all data collection.

In both cases all data was exported as .csv files with the data all fully presented in the main text and supporting files.

Timing and spatial scale

The detailed stratigraphic and chronological information for the five sites studied (Supplementary Note 1) has enabled division of the human and faunal samples into occupation 'Phases' at each site (Figs. 2-6). To display and compare our data on a broader scale we have also divided the human and faunal data into broader island 'Phases' of occupation for Timor and Alor, respectively, based on the stratigraphic and chronometric information noted above (Supplementary Note 1, Supplementary Tables 6-7) (Fig. 2). For Timor, and the sites of Asitau Kuru, Lene Hara, Matja Kuru 1, and Matja Kuru 2 (Timor-Leste), this system includes four broad phases: a Late Pleistocene pre-LGM phase (46,000-21,001 years ago), a Terminal Pleistocene phase (21,000-11,001 years ago); an Early and Middle Holocene phase (11,000-4,001 years ago; and a Late Holocene 'Neolithic' Phase (4000-0 years ago). For Alor, and the sites of Makpan and Tron Bon Lei, the phasing system includes three broad phases: a Late Pleistocene pre-LGM phase (40,000-21,001 years ago), a Terminal Pleistocene to Middle Holocene phase (15,000-7,500 years ago); and a Late Holocene 'Neolithic' Phase (4000-0 years ago). The specific associated dates for the human samples within these broader phases are discussed in the main text where appropriate.

All sampling and data collection was performed between 2017 and 2019. Enamel powder samples were obtained during two research trips by PR to ANU (during 2017 and 2019) where he was assisted by JL, SO'C, SSC, SK, and SH in animal and human tooth identification. The resulting enamel powder was pretreated and analyzed at the Max Planck Institute for the Science of Human History in various runs on the Thermo Gas Bench 2 connected to a Thermo Delta V Advantage Mass Spectrometer between 2017 and 2019. Each tooth identified was sampled once to preserve material for future research.

Data exclusions

No data was excluded from the analyses.

Reproducibility

For the stable isotope analysis Replicate analysis of MERCK standards suggests that machine measurement error is c. ± 0.1‰ for δ13C and ± 0.2‰ for δ18O overall measurement precision was studied through the measurement of repeat extracts from a bovid tooth enamel standard (n = 20, ± 0.2‰ for δ13C and ± 0.3‰). Each sample was run only once to preserve material for future research, as is standard for this type of analysis. However, replicate analysis of the in-house bovid standard (n=20) provides an idea into overall reproducibility (± 0.2‰ for δ13C and ± 0.3‰ for δ18O). Each δ13C and δ18O measurement on the Thermo Gas Bench 2 connected to a Thermo Delta V Advantage Mass Spectrometer at MPI-SHH is the product of 10 measurements of 10 pulses of gas produced from each sample during reaction with the phosphoric acid.

For the FTIR, the reproducibilities of the indices BPI, API, BAI, and PCI are ±0.01, ±0.004, ±0.1 and ±0.1, respectively. Each data point is based on triplicate analysis.

Randomization

Existing, including published, stratigraphic, zooarchaeological, and chronological information was compiled in order to enable the placement of samples in their proper context (island, site, species, date). These categories formed the basis for the hypothesis testing and statistical analyses which is fully reported in the main text paper. Randomization is not relevant for this study as it is based on all available human and animal teeth from archaeological excavations. Therefore the sample size is dictated by preservation and zooarchaeological identification.

Blinding

Blinding is not relevant as the experiments performed are based on all available material. In the case of stable carbon and oxygen isotope analysis all measurement data and calibration have been reported. These materials can also be freely accessed.

Did the study involve field work? ☐ Yes ☒ No

Reporting for specific materials, systems and methods

We require information from authors about some types of materials, experimental systems and methods used in many studies. Here, indicate whether each material, system or method listed is relevant to your study. If you are not sure if a list item applies to your research, read the appropriate section before selecting a response.

Materials & experimental systems

n/a	Involved in the study
☒	☐ Antibodies
☒	☐ Eukaryotic cell lines
☒	☐ Palaeontology
☒	☐ Animals and other organisms
☒	☐ Human research participants
☒	☐ Clinical data

Methods

n/a	Involved in the study
☒	☐ ChIP-seq
☒	☐ Flow cytometry
☒	☐ MRI-based neuroimaging
