# Supplementary material for: Isotopic evidence for initial coastal colonization and subsequent diversification in the human occupation of Wallacea
Source: Nat Commun. 2020 Apr 29;11:2068. doi: 10.1038/s41467-020-15969-4 (PMC7190613; doi:10.1038/s41467-020-15969-4)
Supplement: Supplementary file 4 — Description of Additional Supplementary Files [file 41467_2020_15969_MOESM4_ESM.pdf]

Description of Additional Supplementary Files

Supplementary Data 1. Stable carbon ($\delta^{13}C$) and oxygen ($\delta^{18}O$) isotope data from terrestrial and marine animal tooth enamel samples analyzed in this study (Tab 1: Alor (Makpan, Tron Bon Lei), Tab 2: Timor-Leste (Asitau Kuru, Matja Kuru 2). Provided as Excel Format for ease of access and duplication. In each case it is indicated which site phase and broader regional phase each sample falls into based on discussion in Supplementary Note 1.

Supplementary Data 2. Stable carbon ($\delta^{13}C$) and oxygen ($\delta^{18}O$) isotope data from human tooth enamel samples analyzed in this study (Tab 1: Alor (Makpan, Tron Bon Lei), Tab 2: Timor-Leste (Asitau Kuru, Matja Kuru 1, Matja Kuru 2, Lene Hara). Provided as Excel Format for ease of access and duplication. In each case it is indicated which site phase and broader regional phase each sample falls into based on discussion in Supplementary Note 1.

Supplementary Data 3. Samples selected for in-depth FTIR analysis across the different 'Phases' and sites analyzed. Results for the parameters API, BPI, WAMPI, PCI, and BAI are shown (as per Roche et al. [36]).
